# Heparin induces α-synuclein to form new fibril polymorphs with attenuated neuropathology

**DOI:** 10.1038/s41467-022-31790-7

**Published:** 2022-07-22

**Authors:** Youqi Tao, Yunpeng Sun, Shiran Lv, Wencheng Xia, Kun Zhao, Qianhui Xu, Qinyue Zhao, Lin He, Weidong Le, Yong Wang, Cong Liu, Dan Li

**Affiliations:** 1 Bio-X Institutes, Key Laboratory for the Genetics of Developmental and Neuropsychiatric Disorders (Ministry of Education), Shanghai Jiao Tong University, Shanghai, 200030 China; 2 School of Life Sciences and Biotechnology, Shanghai Jiao Tongś University, Shanghai, 200030 China; 3 Interdisciplinary Research Center on Biology and Chemistry, Shanghai Institute of Organic Chemistry, Chinese Academy of Sciences, Shanghai, 201210 China; 4 University of the Chinese Academy of Sciences, Beijing, 100049 China; 5 Institute of Neurology, Sichuan Academy of Medical Sciences-Sichuan Provincial Hospital, Chengdu, 610072 China; 6 College of Life Sciences, Institute of Quantitative Biology, Zhejiang University, Hangzhou, 310027 China; 7 The Provincial International Science and Technology Cooperation Base on Engineering Biology, International Campus of Zhejiang University, Haining, 314400 China; 8 Bio-X-Renji Hospital Research Center, Renji Hospital, School of Medicine, Shanghai Jiao Tong University, Shanghai, 200240 China; 9 Zhangjiang Institute for Advanced Study, Shanghai Jiao Tong University, Shanghai, 200240 China

**Keywords:** Biophysical chemistry, Cryoelectron microscopy, Protein aggregation, Parkinson's disease

## Abstract

α-Synuclein (α-syn), as a primary pathogenic protein in Parkinson’s disease (PD) and other synucleinopathies, exhibits a high potential to form polymorphic fibrils. Chemical ligands have been found to involve in the assembly of α-syn fibrils in patients’ brains. However, how ligands influence the fibril polymorphism remains vague. Here, we report the near-atomic structures of α-syn fibrils in complex with heparin, a representative glycosaminoglycan (GAG), determined by cryo-electron microscopy (cryo-EM). The structures demonstrate that the presence of heparin completely alters the fibril assembly via rearranging the charge interactions of α-syn both at the intramolecular and the inter-protofilamental levels, which leads to the generation of four fibril polymorphs. Remarkably, in one of the fibril polymorphs, α-syn folds into a distinctive conformation that has not been observed previously. Moreover, the heparin-α-syn complex fibrils exhibit diminished neuropathology in primary neurons. Our work provides the structural mechanism for how heparin determines the assembly of α-syn fibrils, and emphasizes the important role of biological polymers in the conformational selection and neuropathology regulation of amyloid fibrils.

## Abstract (web summary)

The Cryo-EM structures reported in this work reveal how heparin incorporates into α-syn fibril formation to determine fibril polymorphs. This highlights the role of biological polymers in the conformational selection and neuropathological regulation of amyloid fibrils.

## Introduction

α-Synuclein (α-syn) is a key pathogenic protein involved in Parkinson’s disease (PD) and other synucleinopathies^1,2^. It is a small highly-charged protein mainly expressed in neurons and is involved in normal functions such as neurotransmission^3^. α-Syn is innately unstructured and forms various conformations depending on the cellular context. It remains unfolded in the cytosol or forms an α-helix structure in association with cell membranes^4,5^. Under pathological conditions, it undergoes polymerization via non-covalent intermolecular interactions such as hydrogen bonding and π-π stacking, to form β-strand-rich amyloid fibrils^6,7^. These fibrils are toxic to neurons, deposition of which in pathological inclusions is a pathological hallmark of synucleinopathies such as PD, multiple system atrophy (MSA) and dementia with Lewy bodies (DLB)^8^.

Similar to prion proteins, α-syn has been indicated to form different conformational fibrils (strains) in different synucleinopathies^7,9–14^, and the α-syn fibrils also spread in a prion-like manner in the brain^15–17^. Consistently, high-resolution structural determination of α-syn fibrils has unveiled a variety of polymorphic structures of either in vitro fibrils or ex vivo fibrils extracted from the brain of patients^7,18–23^. Notably, the ex vivo fibrils purified from patients with MSA exhibit modification on the amino-acid side chains via covalent posttranslational modifications (PTMs) as well as noncovalently bound with unidentified molecular ligands^7^. Chemical modification has also been widely observed in Tau fibrils purified from patients with various tauopathies^24,25^. Although the chemical modifications of the pathological fibrils have not been well identified yet, they are apparently highly associated with the formation and pathology of the pathological fibrils.

Glycosaminoglycans (GAGs) are integral components of the extracellular matrix, which is involved in the cellular uptake of amyloid fibril seeds during the spread of fibrils in the brain^26–28^. GAGs have also been found in amyloid deposits such as Lewy bodies, and are known to influence amyloid aggregation^29,30^. For example, heparin, a representative GAG, can stimulate the amyloid aggregation of α-syn in vitro^31,32^, but inhibit fibril spreading by blocking the binding of fibril seeds with the cell surface^26^. However, the molecular mechanism by which heparin influences α-syn fibril formation and pathology remains elusive.

In this work, we used cryo-EM to determine the atomic structures of α-syn fibrils that were formed in the presence of heparin and comparatively in the absence of heparin. The structures show that heparin not only presents as a component of the fibril core, but also leads to alternative assemblies of α-syn fibrils, in which a new fold of α-syn was determined. We further show that the heparin-α-syn complex fibrils are less pathological to primary neurons than the apo-fibrils (control fibrils that are formed without heparin). Our work provides mechanistic understanding for the impact of heparin on the structure and pathology of α-syn fibrils, and highlights the potential of biological polymers in the determination of fibril polymorphs.

## Results

### Heparin alters the fibrillar assembly of α-syn

**Fig. 1:**
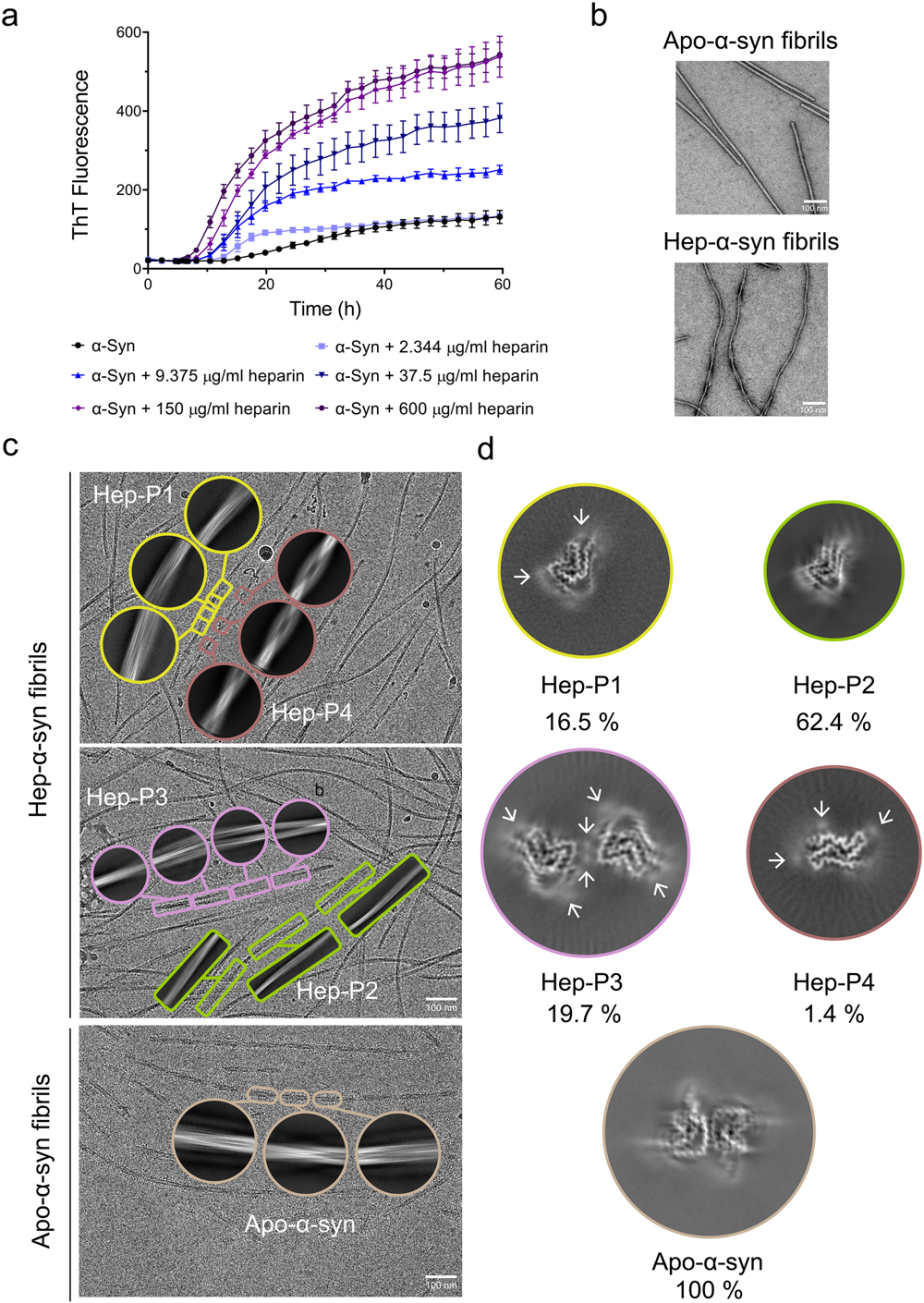
Polymorphic fibrils of α-syn formed in the presence of heparin. **a** ThT kinetic assay for α-syn aggregation in the presence of a gradient concentration of heparin. α-Syn concentration is 50 μM. Data shown are mean ± SD, *n* = 5 independently prepared samples. **b** Representative negative-staining TEM images of apo-α-syn fibrils (top) and hep-α-syn fibrils (bottom) from three biologically independent experiments. Scale bar = 100 nm. **c** Cryo-EM micrographs of hep-α-syn and apo-α-syn fibrils. Representative 2D class averages of each polymorph are shown as insets. Scale bar = 100 nm. **d** Central slices of the 3D maps of each polymorph of hep-α-syn fibrils and apo-α-syn fibrils. The proportions of each polymorph in the fibril sample are indicated. Arrows indicate the additional densities.

To investigate how heparin influences α-syn fibrillation, we began with the thioflavin T (ThT) kinetic assay and morphological characterization by negative staining transmission electron microscopy (TEM). The results showed that heparin significantly promotes α-syn fibrillation in a dose-dependent manner (Fig. 1a), which is consistent with previous reports^31^. TEM imaging further showed that the fibrils formed in the presence of heparin (referred to as hep-α-syn fibrils) are morphologically distinct from those formed in the absence of heparin (referred to as apo-α-syn fibrils) (Fig. 1b).

To further gain atomic details of the fibrils, we prepared cryo-EM samples of the hep-α-syn and apo-α-syn fibrils for structure determination. The cryo-EM micrographs were acquired at ×22,500 magnification on a 300 kV Titan Krios microscope equipped with the K3 Summit camera. 92,118 fibrils picked from 5473 micrographs were used for the reconstruction of the hep-α-syn fibrils (Table 1). 38,830 fibrils from 2321 micrographs were picked for the apo-α-syn fibril. 2D classification revealed that the hep-α-syn fibrils comprise four different polymorphs (referred to as Hep-P1, -P2, -P3, and -P4, respectively) (Fig. 1c). These polymorphs feature different helical parameters including fibril pitch, twist angle and helical rise (Table 1). Three of them including Hep-P1, P2, and P4 contain a single protofilament; in contrast, Hep-P3 is composed of two protofilaments (Fig. 1d). The conformation of α-syn monomer in Hep-P1, P2, and P3 fibrils appears similar, but the overall conformations of fibrils are different in helical parameters (Fig. 1d, Table 1). These hep-α-syn fibril polymorphs are largely distinct from the apo-α-syn fibril in terms of both the conformation of α-syn monomer and the helical parameters of the overall fibril (Fig. 1d, Table 1). The apo-α-syn fibril replicates that of the α-syn fibril prepared under the same condition reported previously^18^ (Supplementary Fig. 1). Strikingly, additional densities are present surrounding or in the interface of the protofilaments of the hep-α-syn polymorphs (Fig. 1d). These densities are isolated from the polypeptide chain of α-syn and are close to the surface lysine residues of α-syn (see the structure models), indicating the incorporation of heparin into the fibril assembly. In addition, we confirmed that both hep- and apo-α-syn fibrils were formed by full-length α-syn by immunogold labeling TEM (Supplementary Fig. 2). Taken together, these observations indicate that heparin involves in the fibrillar assembly of α-syn, changes the fibril structure and generates multiple polymorphs.

**Table 1:** Statistics of cryo-EM data collection, refinement and validation.

Name	Hep-P1	Hep-P2	Hep-P3	Hep-P4	Apo-α-syn fibrils^a^
PDB ID	7V4A	-	7V4B	7V4C	-
EMDB ID	EMD-31705	-	EMD-31706	EMD-31707	-
Data Collection					
Pixel size (Å)	1.06	1.06	1.06	1.06	1.06
Defocus Range (μm)	−2.0 to −1.0	−2.0 to −1.0	−2.0 to −1.0	−2.0 to −1.0	−2.0 to −1.0
Voltage (kV)	300	300	300	300	300
Camera	K3	K3	K3	K3	K3
Microscope	Titan Krios	Titan Krios	Titan Krios	Titan Krios	Titan Krios
Exposure time (s/frame)	0.097	0.097	0.097	0.097	0.097
Number of frames	32	32	32	32	32
Total dose (e^-^/Å^2^)	55	55	55	55	55
Reconstruction					
Micrographs	5473	5473	5473	5473	2321
Manually picked fibrils	92,118	92,118	92,118	92,118	38,830
Box size (pixel)	610	686	398	166	288
Inter-box distance (Å)	64.66	72.72	42.19	17.60	30.53
Segments extracted	83,529	291,650	262,084	188,227	583,394
Segments after Class2D	60,383	157,139	139,930	184,245	227,177
Segments after Class3D	20,111	94,743	47,063	38,286	105,423
Resolution (Å)	3.2	-	3.1	3.4	3.5
Map sharpening B-factor (Å^2^)	−95.39	-	−100.97	−100.35	−105.77
Half pitch (nm)	64.3	140.2	161.2	28.2	128.1
Helical rise (Å)	4.83	4.80	2.42	4.80	2.42
Helical twist (°)	−1.35	−0.62	179.73	−3.06	179.66
Atomic model					
Non-hydrogen atoms	1653	-	3336	1287	-
Protein residues	240	-	480	186	-
Ligands	0	-	0	0	-
r.m.s.d. Bond lengths	0.002	-	0.006	0.008	-
r.m.s.d. Bond angles	0.518	-	0.597	0.745	-
All-atom clash score	8.54	-	15.32	11.74	-
Rotamer outliers	0.00%	-	0.00%	0.00%	-
Ramachandran Outliers	0.00%	-	0.00%	0.00%	-
Ramachandran Allowed	7.89%	-	13.16%	20.00%	-
Ramachandran Favored	92.11%	-	86.84%	80.00%	-

^a^Same structure as a previously reported α-syn fibril structure (PDB ID: 6A6B)^18^ based on the density map.

### Structures of the Hep-α-syn fibril polymorphs P1, P2 and P3

**Fig. 2:**
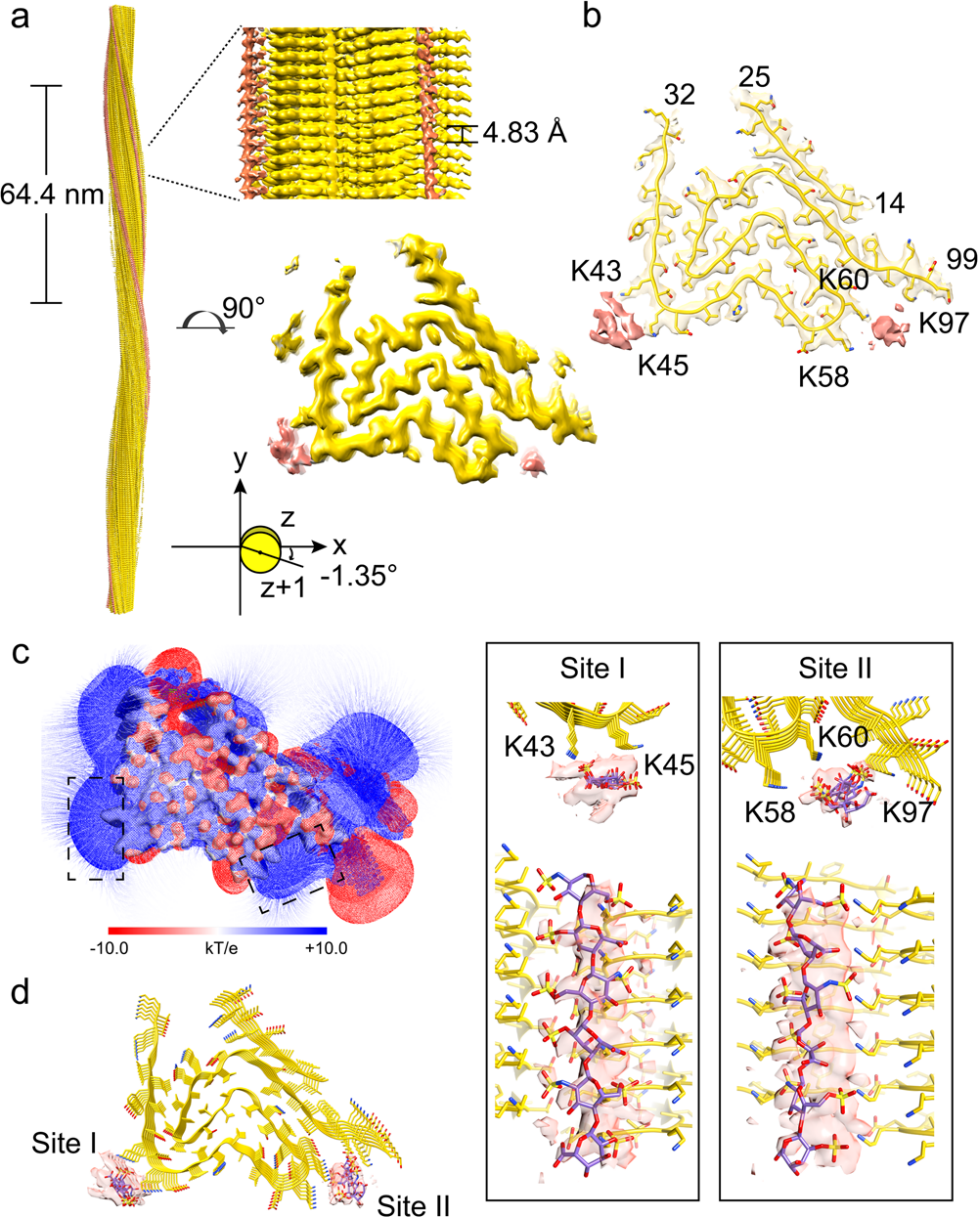
Cryo-EM structure of the Hep-P1 fibril. **a** Cryo-EM density map of the Hep-P1 fibril. The fibril parameters including the length of half pitch (180° helical turn), helical rise and twist angle are indicated. Extra densities are displayed with the same threshold (0.0125) as protein densities. Extra densities for heparin are colored in red in the density map. No specified radius was used to plot around residues/heparin. **b** Cross-section view of the structural model fitted in the density map. The density map is the same map in (**a**) but restricted to areas within a 2 Å radius of the α-syn model, and then combined with the heparin densities in (**a**). **c** Cross-section view of the electrostatic potential surface of the Hep-P1 fibril. The heparin-binding sites are highlighted dash boxes. A surface potential color bar is shown. **d** Molecular docking of heparin into the extra densities. Heparin molecules are shown in sticks and colored in purple). Cross-section view is shown on the left. Side view is shown in boxes on the right.

The overall resolutions of the cryo-EM density maps of Hep-P1 and P3 are 3.2 Å and 3.1 Å, respectively (Supplementary Fig. 3 and Table 1). According to the density maps, we unambiguously built the structural models for Hep-P1 and Hep-P3. Hep-P2 is highly similar to Hep-P1 in terms of the conformation of α-syn monomer, and the resolution of its density map is lower than that of Hep-P1. We thus did not build a structural model for Hep-P2. Hep-P1 contains a single protofilament and features a left-handed helix with a half pitch of 64.4 nm (Fig. 2a, Supplementary Fig. 3). Notably, there are two additional densities observed in adjacent to the positively charged surfaces extended along the fibril axis: one is formed by Lys43 and Lys45, and the other is formed by Lys58, Lys60 and K97 (Supplementary Fig. 4, Fig. 2b, c), which indicates the negatively charged heparin may accommodate in these two positions.

We next performed molecular docking of heparin molecules into the two densities along the fibril axis. The heparin molecules can be well fitted into the density (Fig. 2d). On the Lys43-Lys45 surface (site I), the sulfonic acid group of heparin forms a salt bridge with Lys43 of α-syn. The distance between sulfonic acid groups of heparins and the side chains of Lys43 range from 3.70 to 4.23 Å along the fibril axis. In the Lys58-Lys60-Lys97 binding surface (site II), the sulfonic acid groups of heparin forms salt bridges with Lys58, Lys60, and Lys97 of α-syn (Fig. 2d). Note that the envelopes of the extra densities appear to be larger than the size of the heparin molecules from docking, which suggests that there may be alternative conformations of binding.

The overall architecture of the Hep-P1 fibril core is similar to that of the protofilament in polymorphs 2a and 2b reported previously^20^ (Supplementary Fig. 5A). However, in polymorph 2a or 2b, instead of being stabilized by heparin, Lys45 forms a salt bridge with Glu57 (polymorph 2a) or Glu46 (polymorph 2b) of the neighbouring subunit leading to the pairing of two intertwining protofilaments. Thus, heparin blocks the interaction between Lys45 and Glu57/Glu46 so as to maintain a single protofilamental polymorph of Hep-P1 (Fig. 2d, Supplementary Fig. 5B).

**Fig. 3:**
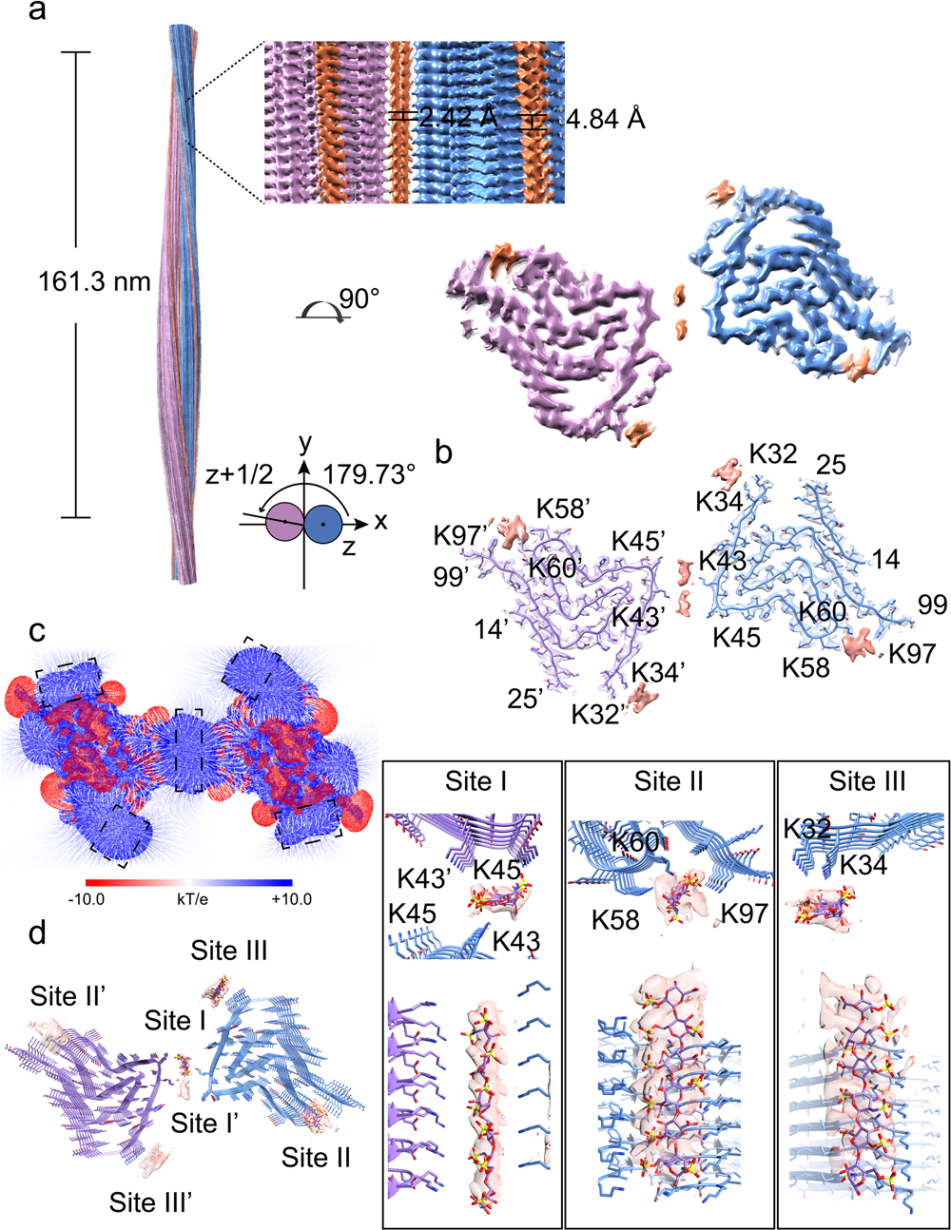
Cryo-EM structure of the Hep-P3 fibril. **a** Cryo-EM density map of the Hep-P3 fibril. The fibril parameters including the length of half pitch (180° helical turn), helical rise and twist angle are indicated. The two protofilements are colored in blue and purple, respectively. Extra densities are displayed with the same threshold (0.008) as protein densities. Extra densities for heparin are colored in orange in the density map. No specified radius was used to plot around residues/heparin. **b** Cross-section view of the structural model fitted in the density map. The density map is the same map in (**a**) but restricted to areas within a 2 Å radius of the α-syn model, and then combined with the heparin densities in (**a**). **c** Cross-section view of the electrostatic potential surface of the Hep-P3 fibril. The heparin-binding sites are highlighted dash boxes. A surface potential color bar is shown. **d** Molecular docking of heparin into the extra densities. Heparin molecules are shown in sticks and colored in purple). Cross-section view is shown on the left. Side view is shown in boxes on the right.

On the other hand, heparin bound to the Lys43-Lys45 surface can also mediate the dimerization of Hep-P1 to form the Hep-P3 polymorph (Fig. 3a, b, Supplementary Fig. 4). Each protofilament of Hep-P3 is highly similar to Hep-P1 including the conformation of α-syn and the binding positions of heparin molecules (Figs. 2d, 3b–d and Supplementary Fig. 5A). In the protofilamental interface of Hep-P3, two heparin molecules are sandwiched by two pairs of Lys43 and Lys45 (Fig. 3d and Supplementary Fig. 5B). Taken together, heparin can not only stabilize α-syn fibrils as a single protofilament as seen in Hep-P1 but also mediate the protofilamental interaction to further assemble into thicker fibrils as seen in Hep-P3.

### A distinctive fold of α-syn in polymorph Hep-P4

**Fig. 4:**
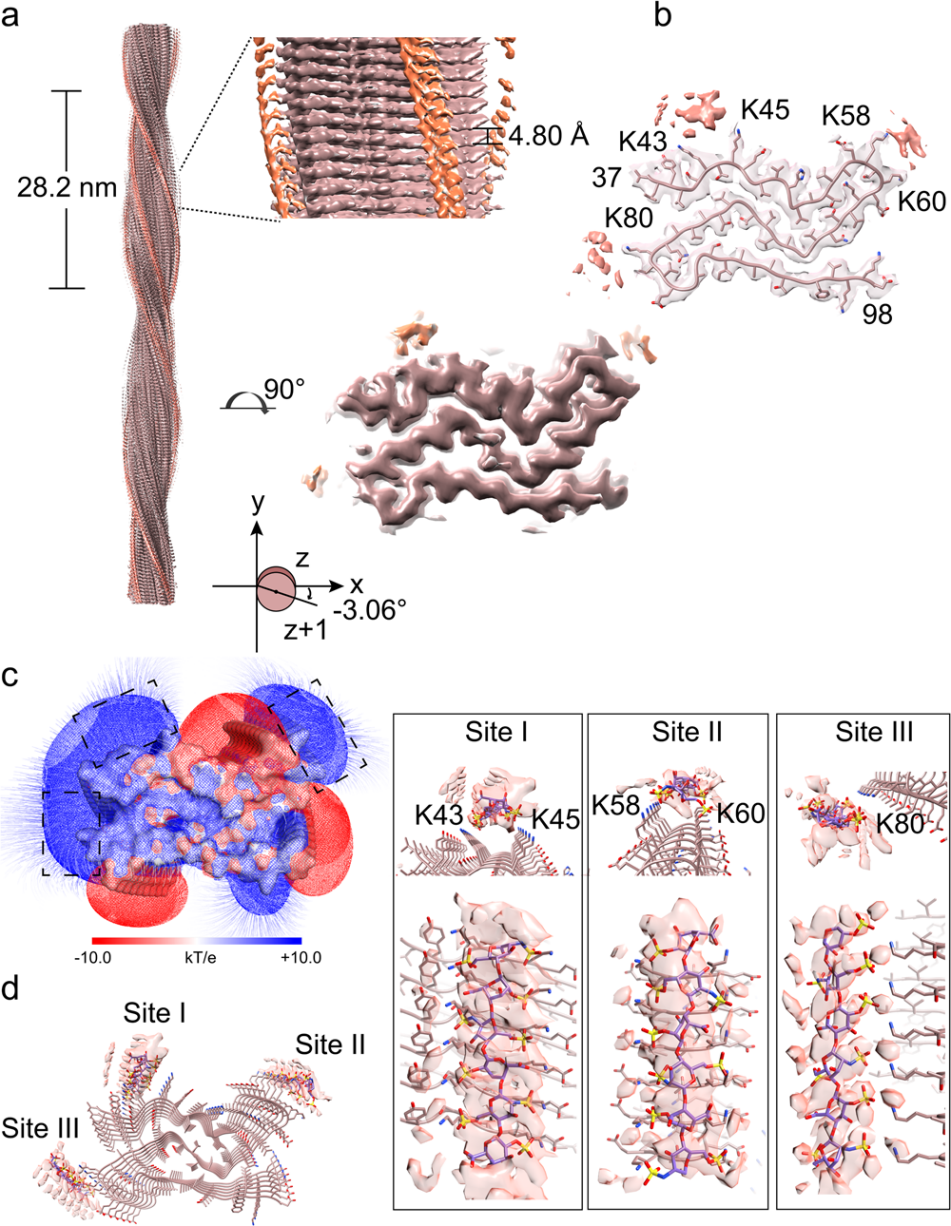
Cryo-EM structure of the Hep-P4 fibril. **a** Cryo-EM density map of the Hep-P4 fibril. The fibril parameters including the length of half pitch (180° helical turn), helical rise and twist angle are indicated. Extra densities are displayed with the same threshold (0.0095) as protein densities. Extra densities for heparin are colored in orange in the density map. No specified radius was used to plot around residues/heparin. **b** Cross-section view of the structural model fitted in the density map. The density map is the same map in (**a**) but restricted to areas within a 2 Å radius of the α-syn model, and then combined with the heparin densities in (**a**). **c** Cross-section view of the electrostatic potential surface of the Hep-P1 fibril. The heparin-binding sites are highlighted dash boxes. A surface potential color bar is shown. **d** Molecular docking of heparin into the extra densities. Heparin molecules are shown in sticks and colored in purple). Cross-section view is shown on the left. Side view is shown in boxes on the right.

The overall resolution of the cryo-EM density map of Hep-P4 is 3.4 Å (Fig. 4a, Supplementary Fig. 3, Table 1). According to the density maps, we built the structural model for Hep-P4. Hep-P4 contains a single protofilament with a half pitch of 28.2 nm (Fig. 4a), which is the shortest among all the α-syn fibril structures reported so far^7,18–23,33–42^. The fibril core of Hep-P4 is composed of residues 37–98 which arranges into a “Z” fold (Fig. 4a, b), which is distinctive from all known α-syn fibril structures reported so far. Three additional density blobs were identified close to the positively charged surface of the Hep-P4 fibril core (Fig. 4a–c, Supplementary Fig. 4), indicating heparin molecules involved in the Hep-P4 fibril formation. Molecular docking data revealed that heparin molecules bind to sites K43-K45 and K58-K60, similar to the heparin binding pattern in the Hep-P1 structure (Fig. 2d). In contrast, K80 which is buried inside the fibril core of Hep-P1 is now exposed to the solvent and serves as a third heparin binding site (Fig. 4d and Supplementary Fig. 6). The binding of heparin with K80 blocks the wrap-over of the N-terminus leading to the open up of the V-shaped structural motif formed by residues 68–79 (Supplementary Fig. 6). Interestingly, the C-terminal half of Hep-P4 containing residues 73–98 forms a structural motif highly similar to that in the so-called type 3 polymorph determined from the fibrils seeded with the cerebrospinal fluid (CSF) of a postmortem patient with PD^42^ (Supplementary Fig. 7A). Such a similar motif also shares some similarity with that in the in vitro polymorph 1a^18^, and ex vivo fibril polymorphs purified from patients with MSA^7^ (Supplementary Fig. 7A). In contrast, the N-terminal half of Hep-P4 containing residues 37–72 forms a structural motif nearly identical to that in Hep-P1, as well as that in polymorphs 2a and 2b^20^ (Supplementary Fig. 7B). Thus, Hep-P4 appears as a hybrid of two common structural motifs seen in the known α-syn fibril structures.

### Diminished neuropathology of heparin-α-syn complex fibrils

Finally, we sought to examine the neuropathology of these heparin-bound polymorphs of α-syn fibrils. A well-established neuronal propagation assay^43^ was performed to monitor the pathological aggregation of endogenous α-syn in primary cortical neurons induced by exogenous preformed α-syn fibrils (PFFs). We applied comparable sizes and amounts of apo- and heparin-α-syn to the primary neurons (Supplementary Fig. 8), as well as a control sample of apo-α-syn PFFs supplemented with heparin in a comparable amount to that in heparin-α-syn PFFs in the medium. Strikingly, compared with the apo-α-syn PFFs, both the heparin-α-syn PFFs and apo-α-syn PFFs supplemented with heparin-induced significantly less pathological α-syn aggregation in neurons immunostained by pS129 α-syn antibody (Supplementary Fig. 9A), which is consistent with previous studies showing that heparin can inhibit the cellular uptake of α-syn fibrils^26,32^.

To confirm the role heparin in the reduced capability of heparin-α-syn PFFs in inducing endogenous α-syn aggregation in neurons, we measured the neuronal surface binding and cellular uptake of the heparin-α-syn PFFs in comparison with the apo-α-syn PFFs. As shown in Supplementary Fig. 9B, the heparin-α-syn PFFs displayed a dramatically decreased ability of neuronal binding compared to the apo-α-syn PFFs. In addition, the neuronal uptake of heparin-α-syn PFFs also decreased compared to that of the apo-α-syn PFFs (Supplementary Fig. 9C).

Since the reduced capability of heparin-α-syn PFFs is attributed to heparin, it is still not known whether the apo- and heparin-α-syn PFFs have different activities in seeding endogenous α-syn, if the endogenous α-syn could be exposed to comparable amounts of these PFFs. Therefore, we performed the ThT kinetic assay in vitro. We used an equal amount of apo- and heparin-α-syn PFFs (0.1% or 0.5%) to seed the aggregation of monomeric α-syn. Remarkably, the result showed decreased seeding activity of the heparin-α-syn PFFs compared to the apo-α-syn PFFs, with a significantly elongated lag time of the seeded aggregation (Supplementary Fig. 10).

We next sought to further examine the seeding capacity of the PFFs in neurons. Given that the binding of heparin would block the cellular uptake of α-syn PFFs, to overcome this problem, we used liposomes to deliver PFFs into the cells (Supplementary Fig. 11A). We performed a gradient level of α-syn PFFs (5 nM, 20 nM, 80 nM, and 200 nM), and the result showed the induction of endogenous α-syn aggregation by PFFs in a dose-dependent manner (Supplementary Fig. 11B, C). More importantly, compared to the apo-α-syn PFFs, the heparin-α-syn PFFs exhibited a significantly weaker seeding activity in inducing aggregation of endogenous α-syn in primary neurons (Supplementary Fig. 11B, C), which is consistent with the result of in vitro ThT assay (Supplementary Fig. 10).

Collectively, these results demonstrate that the heparin-α-syn complex fibrils are less pathogenic than the apo fibrils in the aspects of neuronal binding and uptake, as well as the seeding activity, which indicates that ligand binding can not only alter the structure but also the pathology of amyloid fibrils.

## Discussion

**Fig. 5:**
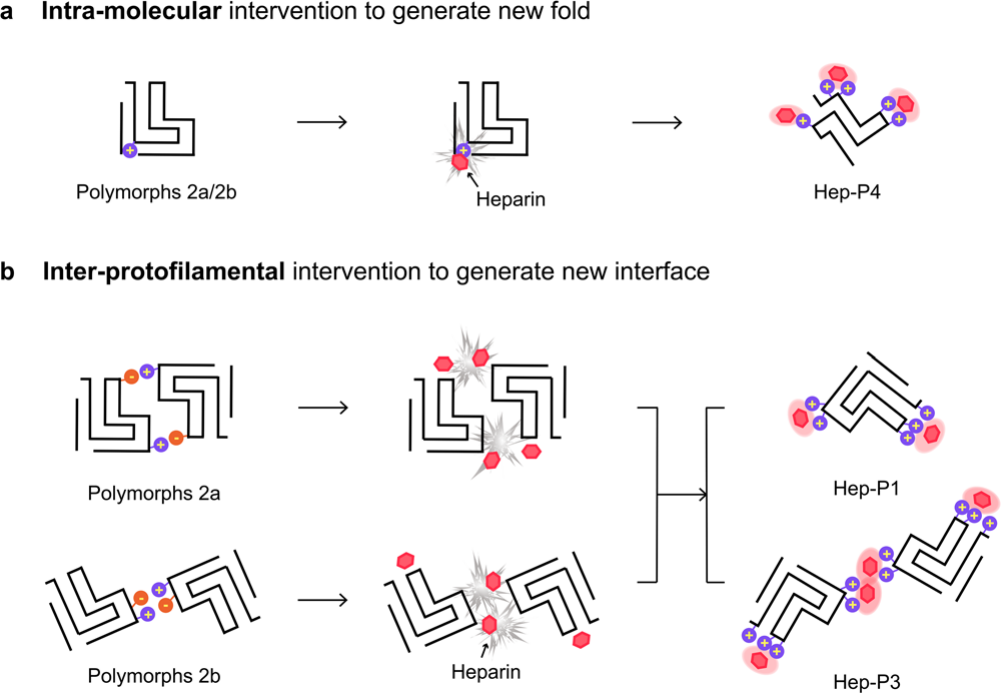
Schematic diagram of the polymorphism of α-syn fibrils induced by heparin. **a** Charge interactions of heparin with lysine residues that are buried inside the polymorphs 2a/2b fold block α-syn folds into polymorphs 2a/2b. Instead, α-syn forms a distinctive fold in complex with heparin. **b** Heparin disrupts the charge interactions between the protofilaments, which results in polymorphs either containing a single protofilament, or forming an interface mediated by heparin.

Structural polymorphism is a unique property of amyloid fibrils, which underlies the pathological heterogeneity of neurodegenerative diseases^34,44,45^. Recent structural studies have revealed several polymorphic fibril structures formed by α-syn under different conditions^25,34^. However, what determines the conformation of α-syn fibrils remains elusive. Cryo-EM structures of α-syn fibrils purified from the brain of patients with MSA unveiled the involvement of chemical modifications including covalent PTM and unidentified non-covalent cofactor binding in the fibril assembly^7^. Similar modifications have also been found in Tau fibrils purified from human tissues^24,25,46,47^. These evidences indicate the important role of non-proteinaceous components in the conformational selection of amyloid fibrils. Notably, the ex vivo α-syn fibrils of MSA contain negatively charged molecules which play an essential role in mediating the interface between protofilaments^7,25^. Unfortunately, these molecules were not able to be identified. However, several negatively charged molecules, e.g., GAGs, indeed have been found to influence α-syn fibril formation^32^. In this work, we showed that heparin, one of the most studied GAGs, can induce α-syn to form special fibril polymorphs by both blocking the original intra- and inter-molecular interactions and establishing new interactions (Fig. 5). Of note, we do not think that these in vitro fibrils could fully replicate the native conformations of α-syn fibrils due to the lack of native modifications. Scheres and colleagues have recently reported that heparin induced Tau to form polymorphic filaments that differ from those in Alzheimer’s and Pick’s diseases^48^. Nevertheless, our work reveals how α-syn polymorphic fibrils could be regulated by noncovalent ligand binding, which not only provides fundamental understanding for the regulation of ligands to the polymorphism of amyloid fibrils, but also provides a strategy to intervene in fibril pathology.

Diamond and colleagues found that heparan sulfate proteoglycans (HSPGs) play an important role in mediating cellular uptake of α-syn and Tau, which can be blocked by the addition of heparin^26^. Consistently, our primary neuron assay showed that the heparin-α-syn complex fibrils exhibit largely attenuated neuropathology (Supplementary Fig. 9). These phenomena can be well explained by the structures of the heparin-α-syn complex fibrils, in which heparin shields available binding sites for HSPGs by either directly binding with lysine residues (K43, K45, K58, K60 and K80) of α-syn, or rearranging the fibril structures. Note that heparin may not only bind with the lysine residues in the fibril core; those N-terminal of the fibril core may also interact with heparin via charged interactions^32^. As a result, HSPGs are not able to well recognize heparin-α-syn fibrils for cell uptake. Of note, in addition to HSPGs, cell surface protein receptors including LAG3 and APLP1 were also found to directly bind with α-syn fibrils^49–51^. However, their mechanisms differ from that of HSPGs. LAG3 and APLP1 recognize the negatively charged C-terminal region of α-syn rather than the lysine residues on the fibril spine^50^. This is consistent with our observation that heparin-α-syn fibrils still maintain some degree of membrane binding and neuronal propagation abilities. Nevertheless, development of heparin-like molecules to efficiently shield the lysine residues on the fibril interface of α-syn may provide a promising strategy for the inhibitor design to prevent the spread of pathological α-syn fibrils. Our structural study may inform rational design of such inhibitors.

## Methods

### Recombinant α-syn monomer expression and purification

The N-terminally acetylated human wild type (WT) α-syn monomer was over-expressed and purified from *E. coli* BL21 (DE3) cells. The gene encoding human full-length WT α-syn was inserted into pET-22b(+) vector and co-transformed with fission yeast N-acetyltransferase complex B in pACYCDuet-1 vector. *E. coli* BL21 (DE3) cells bearing the two plasmids were treated with 1 mM isopropyl-1-thio-D-galactopyranoside (IPTG) at 25 °C for 12 h to induce protein expression. Cells were collected, resuspended in 100 mM Tris-HCl (pH 8.0), 1 mM EDTA, with 1 mM phenylmethylsulfonyl fluoride (PMSF), and lysed through high-pressure cell crusher. After centrifugation at 20,817 × g at 4 °C for 30 min, the supernatant was collected and boiled for 10 min. After centrifugation, 20 mg ml^−1^ streptomycin was added in the supernatant to remove nucleic acids. After a second round of centrifugation, the pH of supernatant was adjusted to 3.5 with 2 M HCl to precipitate other proteins. Then the supernatant was dialyzed in 25 mM Tris-HCl (pH 8.0) at 4 °C overnight. After dialysis, the protein sample was purified sequentially by Q column (GE Healthcare, 17-5156-01) and Superdex 75 column (GE Healthcare, 28-9893-33). For Superdex 75 column, the target protein was eluted with the buffer containing 50 mM Tris-HCl (pH 7.5), 150 mM KCl. The concentration of purified α-syn monomer was determined by Pierce^TM^ bicinchoninic acid (BCA) protein assay kit (ThermoFisher Scientific, 23227). The purity of protein sample was verified by SDS-PAGE.

### ThT kinetic assay

The reaction mixture contained 50 μM α-syn monomer, 50 μM ThT, 0.05% NaN_3_, in 50 mM Tris-HCl (pH 7.5), 150 mM KCl, in the presence of 0–600 μg ml^−1^ heparin (Sigma-Aldrich, H3149) as indicated. Reactions were performed in a black 384-well plate with a clear bottom (ThermoFisher Scientific, 142761). The plate was continuously shaken (900 rpm, orbital) at 37 °C in the BMG FLUOstar Omega plate reader. The fluorescent intensities of each reaction were recorded at excitation of 440 nm and emission of 480 nm every 2 h. The kinetic curves were graphed with GraphPad Prism 8.

### Preparation of apo-α-syn fibrils and hep-α-syn fibrils

Recombinant α-syn monomer (200 μM, in buffer containing 50 mM Tris-HCl, 150 mM KCl, 0.05% NaN_3_, pH 7.5) was continuously shaken at 900 rpm in the absence (for apo-α-syn fibrils) or presence (for hep-α-syn fibrils) of 2.4 mg ml^−1^ heparin in ThermoMixer (Eppendorf). After 5 days agitation, the α-syn fibril samples were sonicated on ice for 30 s (1 s on, 1 s off, 20% amplitude, JY92-IIN sonicator) to obtain seeds. For cryo-EM, negative-staining transmission electron microscopy (TEM), and primary cortical neuronal experiments, α-syn monomer (50 μM, in buffer containing 50 mM Tris-HCl, 150 mM KCl, 0.05% NaN_3_, pH 7.5) was incubated with 1% (molar ratio) preformed fibril seeds in the absence (for apo-α-syn fibrils) or presence (for hep-α-syn fibrils) of 600 μg ml^−1^ heparin with 900 rpm agitation for 5 days. The morphologies of mature apo-α-syn fibrils and hep-α-syn fibrils were validated by negative-staining TEM.

### Negative-staining TEM

A drop of 5 μl fibril samples were loaded onto the glow-discharged 200 mesh carbon support film copper grids (Beijing Zhongjingkeyi Technology Co., Ltd.). After incubations of 45 s, the grids were washed with 5 μl double-distilled water followed by 3% uranyl acetate. Then, the grids were stained with 3% uranyl acetate for another 45 s. Finally, the grids were dried in air and applied to a Tecnai T12 microscope (FEI Company) operated at 120 kV for negative staining TEM imaging.

### Immunogold labeling

200 mesh carbon support film copper grids (Beijing Zhongjingkeyi Technology Co., Ltd.) were glow discharged for 45 s before sample preparation. 5 µL of 10 µM fibril sample solutions were applied to the grids and incubated for 2 min, then excess sample solution were blotted off using the filter paper. Then, blocking buffer containing 0.1% (w/v) BSA in PBS, pH 7.4 was applied to the grids and incubated for 10 min, excess solution was blotted off. Primary antibodies specific for N-terminus (Syn303, Cat.# MMS-5085, BioLegend, mouse monoclonal IgG, epitope residues 1–5) or C-terminus of α-syn (Cat.# ab138501, abcam, rabbit monoclonal IgG, epitope residues 118–123) diluted 1:100 in blocking buffer was applied to the grids and incubated for 2 h, excess solution was blotted off. Control grids were applied in the absence of α-syn antibodies. The grids were washed once with PBS, and excess liquid was blotted off. After that, 12 nm Colloidal Gold-AffiniPure Donkey Anti-Mouse IgG (Jackson ImmunoResearch Laboratories, Inc., Catalog No. 715-205-150) or 12 nm Colloidal Gold-AffiniPure Donkey Anti-Rabbit IgG (Jackson ImmunoResearch Laboratories, Inc., Catalog No. 711-205-152) diluted 1:50 in blocking buffer was applied to the grids and incubated for 1 h, excess solution was blotted off. The grids were washed once with PBS, and excess liquid was blotted off. The grids were negatively stained with 3% uranyl acetate for another 45 s. Finally, the grids were dried in air and applied to a Tecnai T12 microscope (FEI Company) operated at 120 kV for negative staining TEM imaging.

### Cryo-EM data collection

Apo-α-syn fibrils and hep-α-syn fibrils at the concentration of 3 μM (4 μl) were applied to the glow-discharged holey carbon Cu grids (Quantifoil R2/1, 300 mesh) twice and then plunge-frozen in liquid ethane after blotted with filter paper using Vitrobot Mark IV (FEI Company). Cryo-EM micrographs were acquired using a Titan Krios transmission electron microscope (FEI Company) operated at 300 kV with a Gatan K3 direct detector in super-resolution mode. 32 frame movies per micrograph were recorded at × 22,500 magnification with a pixel size 1.06 Å pixel^−1^. The total dose is ~55 e^-^ Å^−2^ for a total exposure time of 3.111 s. For defocus values, a range of −1.0 to −2.0 μm was adopted. Automated cryo-EM data collection was performed by Serial EM 3-7-3 software^52^.

### Image pre-processing, helical reconstruction and model building

For image pre-processing, 32 movie frames per micrograph were corrected for beam induced motion, aligned, dose-weighted, and further binned with a physical pixel size of 1.06 Å using MotionCorr2 1.2.1^53^. The resulting micrographs were used to estimate the contrast transfer function using CTFFIND-4.1.8^54^. Fibrils were manually picked using the “Manual picking” program in RELION 3.1^55^. All subsequent helical reconstruction steps were performed in RELION 3.1^55^.

#### Hep-α-syn fibrils datasets

92,118 manually picked fibrils from 5473 micrographs were extracted to segments with a box size of 1200 pixels and an inter-box distance of 127.2 Å. Then, initial reference-free 2D classification steps with a decreasing in-plane angular sampling rate from 12° to 1° and a T = 2 regularization parameter were performed to separate four polymorphs of hep-α-syn fibrils (termed Hep-P1, -P2, -P3, and -P4, respectively) according to distinct helical crossover distances. The reported ratios of each polymorph were determined by initial 2D classification.

#### Polymorphs of hep-α-syn fibrils

Hep-P1, Hep-P2, Hep-P3, and Hep-P4 fibril segments were re-extracted using box size of 610 pixels, 686 pixels, 398 pixels, and 166 pixels, respectively. For each polymorph, after several iteration of reference-free 2D classification steps with decreasing in-plane angular sampling rate from 12° to 1° and a T = 2 regularization parameter, the purified segments comprising an entire helical crossover were used to calculate the apparent half pitches from the 2D class averages and construct the initial 3D models. These helical parameters and initial 3D models that were low-pass-filtered to 60 Å were further applied to perform 3D classifications (*k* = 1) for each polymorph. Helical symmetry local optimization of helical twist and rise was performed while β-strands perpendicular to the helical axis were clearly separated. The optimized density maps were used for further 3D classifications (k = 3 followed by *k* = 1). 3D auto-refinements with optimization of helical twist and rise after reconstructions were carried out. The final helical rise of Hep-P1, Hep-P2, Hep-P3, and Hep-P4 were optimized to 4.83 Å, 4.80 Å, 2.42 Å, and 4.80 Å, respectively. The final helical twist of Hep-P1, Hep-P2, Hep-P3, and Hep-P4 were optimized to −1.35°, −0.62°, 179.73°, and −3.06°, respectively. For Hep-P1, Hep-P3, and Hep-P4, the maps were further sharpened with a soft-edge solvent mask using the standard post-processing program in RELION 3.1^55^. Overall resolution estimates were calculated based on the gold-standard 0.143 Fourier shell correlation (FSC) between the two independently refined half-maps. Reported overall resolution was 3.2 Å for Hep-P1, 3.1 Å for Hep-P3, and 3.4 Å for Hep-P4, respectively. Local resolution was estimated using the “Local resolution” procedure in RELION 3.1 with the same mask and B-factor in post-processing. Density maps are exhibited via UCSF Chimera 1.13.1.

The atomic models were built into the central region of the sharpened density map de novo in COOT 0.8.9.2^56^. A 3-layer models were generated and refined in the real-space refinement program in PHENIX 1.13^57^. Additional details for helical reconstruction and model building are given in Table 1.

#### Apo-α-syn fibrils dataset

38,830 manually picked fibrils from 2321 micrographs were extracted to segments with a box size of 686 pixels and an inter-box distance of 72.7 Å. Then, reference-free 2D classification steps with a decreasing in-plane angular sampling rate from 12° to 1° and a T = 2 regularization parameter were performed to purify segments and to estimate apparent half pitch. Then, the apo-α-syn fibril segments were re-extracted using a box size of 288 pixels. After several iteration of reference-free 2D classification steps, the purified segments comprising an entire helical crossover were used to generate initial 3D model. The helical parameters and initial 3D model that was low-pass-filtered to 60 Å were further applied to perform 3D classifications (*k* = 1). Helical symmetry local optimization of helical twist and rise was performed while β-strands perpendicular to the helical axis were clearly separated. The optimized density map was used for further 3D classifications (*k* = 3 followed by *k* = 1). 3D auto-refinements with optimization of helical twist and rise after reconstructions was carried out. The final helical parameters were optimized to the rise of 2.42 Å and the helical twist angle of 179.66°. Additional details for helical reconstruction are given in Table 1.

### Molecular docking of heparin

Initial molecular model for heparin was downloaded from Pubchem database (CID: 92044406) and re-modeled as 6-mer. Polar hydrogens were added to the heparin-6-mer ligands as well as the cryo-EM structural models of Hep-P1, Hep-P3, and Hep-P4, respectively, for subsequent molecular docking which was performed via AutoDockTools-1.5.6^58^ and AutoDock Vina 1.2.2^59^. A grid box with a dimension of 34 × 14 × 80 Å^3^ was applied to each fibril structure covering the highly positively charged pockets analyzed using APBS plugin in PyMol 2.0^60^. For each binding site, nine heparin-binding poses were calculated. After multiple rounds of refinements by manual adjustment in PyMol 2.0 guided by the cryo-EM density maps, the heparin-6-mers in complex with hep-α-syn fibril featuring the largest numbers of molecular contacts and low free binding energy were reported.

### Primary cortical neuronal cultures and neuronal assays

Primary cortical neurons were dissected from the cortex of embryonic day (E)15-E17 Sprague-Dawley (SD) rats (commercially obtained from Shanghai SIPPR BK Laboratory Animals Ltd, China) embryos according to a previous described protocol^43^. For immunofluorescence assays, the suspensions of primary cortical neurons were plated in the 24-well plates that pre-covered with poly-D-lysine coated coverslips (150,000 cells/coverslip). For western blot, 500,000 primary cortical neurons were plated in the 12-well plates coated with poly-D-lysine. All rat experiments were performed followed the protocols approved by the Animal Care Committee of the Interdisciplinary Research Center on Biology and Chemistry (IRCBC), Chinese Academy of Sciences (CAS).

For cell treatment, mature fibril samples were centrifuged at 20,817 × g for 1 h at 25 °C. Supernatants were carefully collected, in which the protein concentrations were measured through BCA assay. The quantity of fibrils in the pellets were calculated as the amount of total monomer minus the amount of protein in the supernatant. Then the pellets were washed twice and resuspended in PBS to 100 μM. Sonicated preformed fibrils (PFFs) of apo-α-syn fibrils and hep-α-syn fibrils were prepared by sonicating equal concentrations of resuspended fibrils on ice for 30 s (1 s on, 1 s off, 20% amplitude, JY92-IIN sonicator). Both PFFs were characterized by negative staining TEM to measure the length and to quantify the number in each view. Before cell treatment, both PFFs were confirmed similar in size and number.

#### Neuronal propagation assay

Primary cortical neurons cultured at 10 days (DIV10) were treated with PBS, 200 nM α-syn monomer, 200 nM apo-α-syn PFFs, 200 nM heparin-α-syn PFFs, and 200 nM apo-α-syn PFFs + 2.4 μg ml^−1^ heparin (comparable amount of heparin with that in heparin-α-syn PFFs, mixed in medium) for 14 days (3 biological replicates in each group), respectively. Then neurons were collected and fixed for immunostaining. Primary antibodies composition: anti-p-α-syn (Abcam, Cat.# ab51253, 1:1,000 dilution) and anti-MAP2 (Abcam, Cat.# ab5392, 1:250 dilution). Secondary antibodies composition: Alexa Fluor 488-conjugated goat anti-chicken (ThermoFisher Scientific, Cat.# A11039, 1:1,000 dilution) and Alexa Fluor 568-conjugated goat anti-rabbit (ThermoFisher Scientific, Cat.# A11036, 1:1,000 dilution). The fluorescent images were scanned on a SP8 confocal microscope. The fluorescent gray values of p-α-syn and MAP2 signal were measured by ImageJ 2.0.0.

#### Neuronal surface binding assay

Following the previously established neuronal surface binding assay^50^, primary cortical neurons cultured at 12 days (DIV12) were treated with PBS, 200 nM α-syn monomer, 200 nM apo-α-syn PFFs, and 200 nM hep-α-syn PFFs for 2 h at 25 °C (3 biological replicates in each group), respectively. Then, the neurons were collected, washed, and fixed for immunostaining. Primary antibodies composition: anti-α-syn (Abcam, Cat.# ab138501, 1:1,000 dilution) and anti-MAP2 (Abcam, Cat.# ab5392, 1:250 dilution). Secondary antibodies composition: Alexa Fluor 488-conjugated goat anti-chicken (ThermoFisher Scientific, Cat.# A11039, 1:1,000 dilution) and Alexa Fluor 568-conjugated goat anti-rabbit (ThermoFisher Scientific, Cat.# A11036, 1:1,000 dilution). The fluorescent images were scanned on a SP8 confocal microscope. The fluorescent gray values of α-syn and MAP2 signal were measured by ImageJ 2.0.0.

#### Neuronal uptake assay

Primary cortical neurons cultured at 12 days (DIV12) were treated with PBS, 400 nM α-syn monomer, 400 nM apo-α-syn PFFs, and 400 nM hep-α-syn PFFs for 3 h at 37 °C (3 biological replicates in each group), respectively. Then neurons were washed three times with PBS and precipitated with 10% trichloroacetic acid (w/v) in PBS for 30 min on ice, followed by centrifugation at 20,817 × g at 4 °C for 10 min. The resulting pellets were washed by ice acetone twice, and dissolved in SDS-PAGE sample buffer (0.125 M Tris-HCl, 4% [w/v] SDS, 20% [v/v] glycerol, and 0.01% [w/v] bromophenol blue) by boiling for preparation of the whole-cell lysates. The α-syn contents in cell lysates were immunoblotted by anti-α-syn (Abcam, Cat.# ab138501, 1:1,000 dilution) and normalized to the expression level of β-actin (Cell Signaling Technology, Cat.# 3700 S, 1:1000 dilution).

### Seeding amplification assay

To compare the seeding property of the comparable amount of apo-α-syn PFFs and heparin-α-syn PFFs by ThT kinetic assay. Nonlinear regression was used to fit each kinetic curves to a sigmoidal four parameters dose-response (variable slope) equation in GraphPad Prism 8, and then lag times were calculated using the following equation: Lag time = *T*_50_ – [1/(2×*k*)], where *T*_50_ is the time at which fluorescence is halfway between the baseline and plateau values, and *k* is the Hill slope. The fitting also obtained max fluorescence intensity.

### Liposome mediated seeds transduction

To test the seeding potential of apo-α-syn PFFs and heparin-α-syn PFFs to endogenous α-syn, a previously published protocol was adopted^61,62^. Briefly, equal volumes of Lipofectamine-2000 (Invitrogen) and α-syn PFFs solutions, both diluted in Neurobasal Medium (Thermo Fisher) without B27 supplement and Penicillin-Streptomycin, were combined to give final concentrations of 0.4% (v/v) Lipofectamine-2000 and 5, 20, 80, 200 nM total α-syn, respectively, and then incubated for 20 min to allow liposome formation. Transduction mixture (100 μL per well) was added to primary cortical neurons plated in the 24-well plates that pre-covered with poly-D-lysine coated coverslips (150,000 cells/coverslip). After incubating for 24 h, the transduction medium was then removed and replaced with fresh Neurobasal Medium (Thermo Fisher) for an additional 7 days.

### Reporting summary

Further information on research design is available in the Nature Research Reporting Summary linked to this article.

## Supplementary information

Supplementary information

Reporting Summary

## Data Availability

Density maps of the Hep-P1, Hep-P3, and Hep-P4 fibrils have been deposited in the Electron Microscopy Data Bank (EMDB) under the accession codes EMD-31705 (Hep-P1), EMD-31706 (Hep-P3), and EMD-31707 (Hep-P4), respectively. The structural models have been deposited in the Protein Data Bank (PDB) under the accession codes 7V4A (Hep-P1), 7V4B (Hep-P3), and 7V4C (Hep-P4), respectively. The structural models used in this study are available in the PDB database under accession codes 6A6B (polymorph 1a), 6SSX (polymorph 2a), 6SST (polymorph 2b), and 6XYO (MSA PF-IA), 7V49 (CSF seeded type 3 polymorph) respectively. Other data that support the findings of this study are available from the corresponding author upon reasonable request. Source data are provided with this paper.

## Source data

Source Data
