# Supplementary material for: Heparin induces α-synuclein to form new fibril polymorphs with attenuated neuropathology
Source: Nat Commun. 2022 Jul 22;13:4226. doi: 10.1038/s41467-022-31790-7 (PMC9307803; doi:10.1038/s41467-022-31790-7)
Supplement: Supplementary file 1 — Supplementary information [file 41467_2022_31790_MOESM1_ESM.pdf]

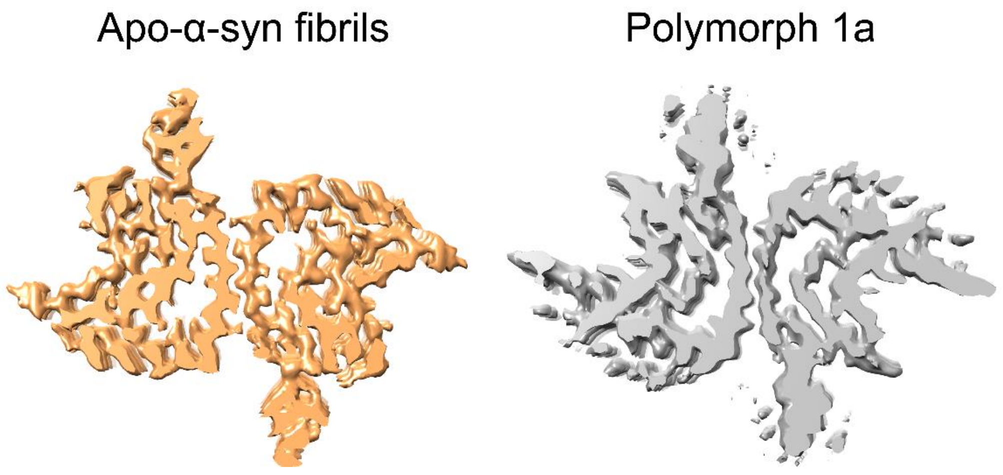

Supplementary Figure 1. Density maps of the apo-α-syn fibril and α-syn fibril polymorph 1a.

Cross-section view of the density maps of the apo-α-syn fibril (left) and α-syn fibril polymorph 1a (PDB ID: 6A6B, EMDB ID: EMD-6988).

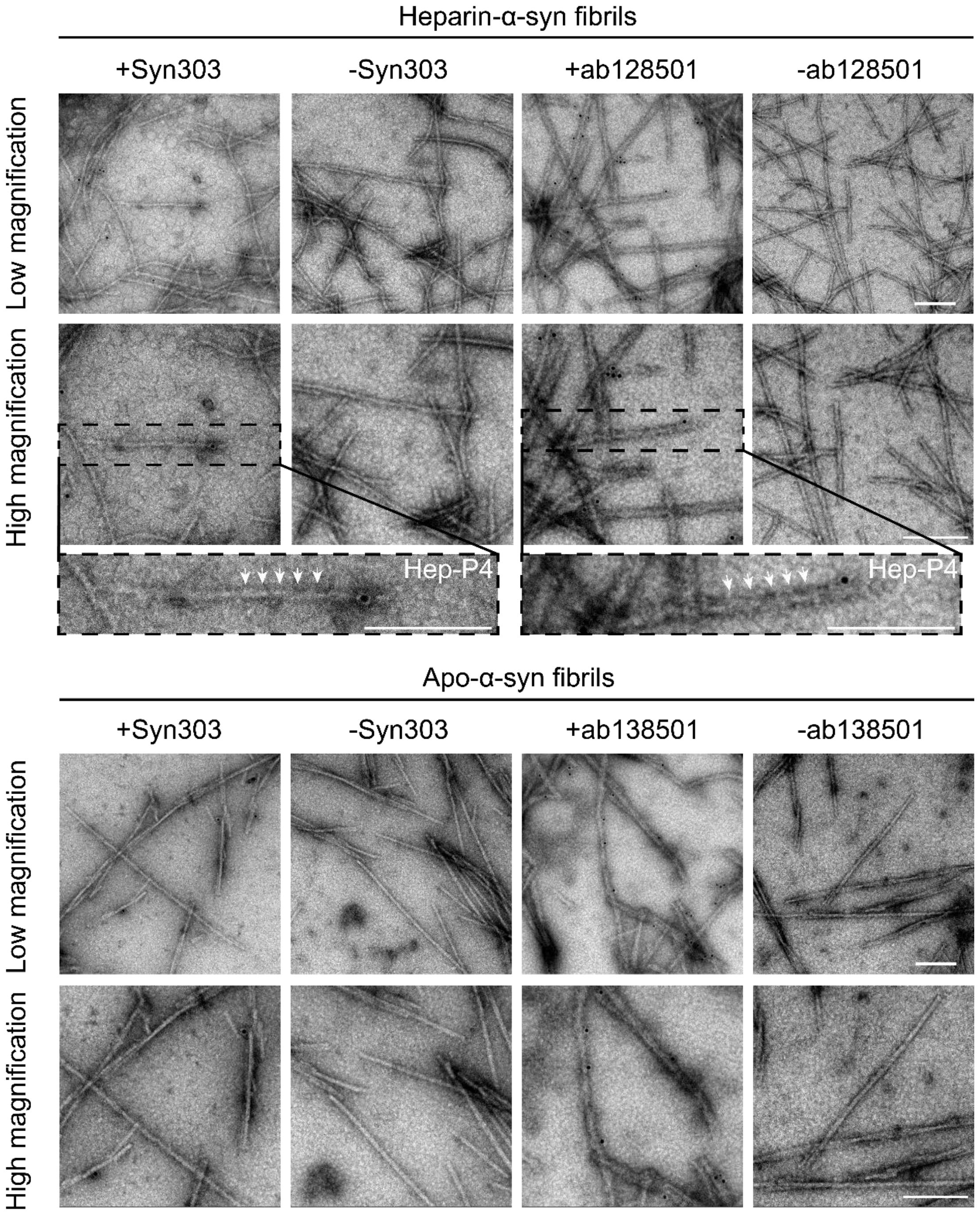

Supplementary Figure 2. Immunoglod labeling TEM of α-syn in fibrils. Representative immunogold labeling TEM imaging of heparin- and apo-α-syn fibrils. The fibrils were incubated with α-syn antibodies Syn303 (recognizing residues 1-5) and ab138501 (recognizing residues 118-123) from three biologically independent experiments. Scale bar, 200 nm. Hep-P4 in the heparin-α-syn fibrils is enlarged. White arrows indicate the helical half pitches of Hep-P4.

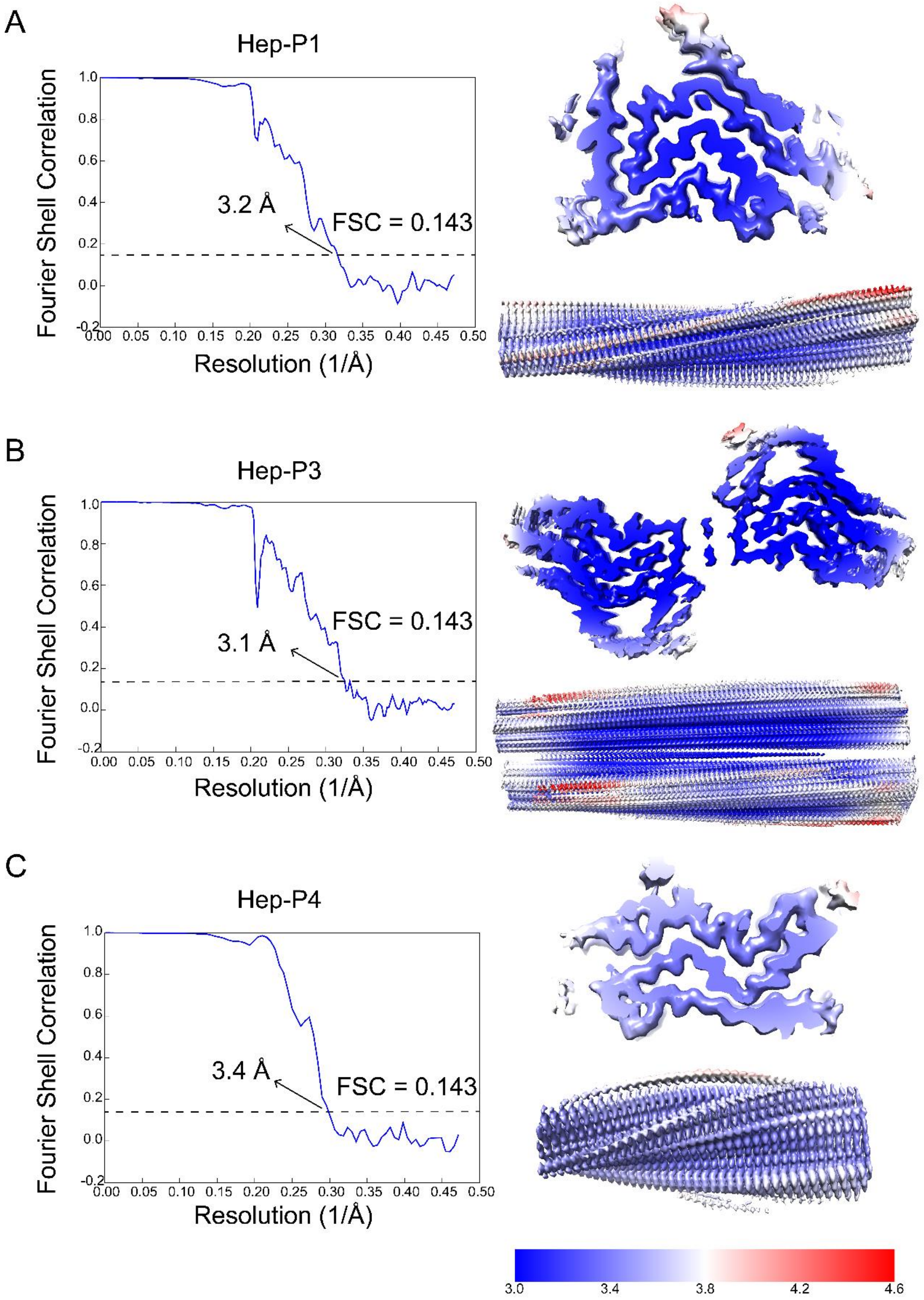

Supplementary Figure 3. Resolution estimation of the cryo-EM structures of Hep-P1, Hep-P3 and Hep-P4.

Left: Gold standard Fourier shell correction curves for the Hep-P1 (**A**), Hep-P3 (**B**), and Hep-P4 (**C**). The overall resolution of Hep-P1, Hep-P3, and Hep-P4 is 3.2 Å, 3.1 Å, and 3.4 Å, respectively.

Right: Local resolution estimation for the Hep-P1 (**A**), Hep-P3 (**B**), and Hep-P4 (**C**).

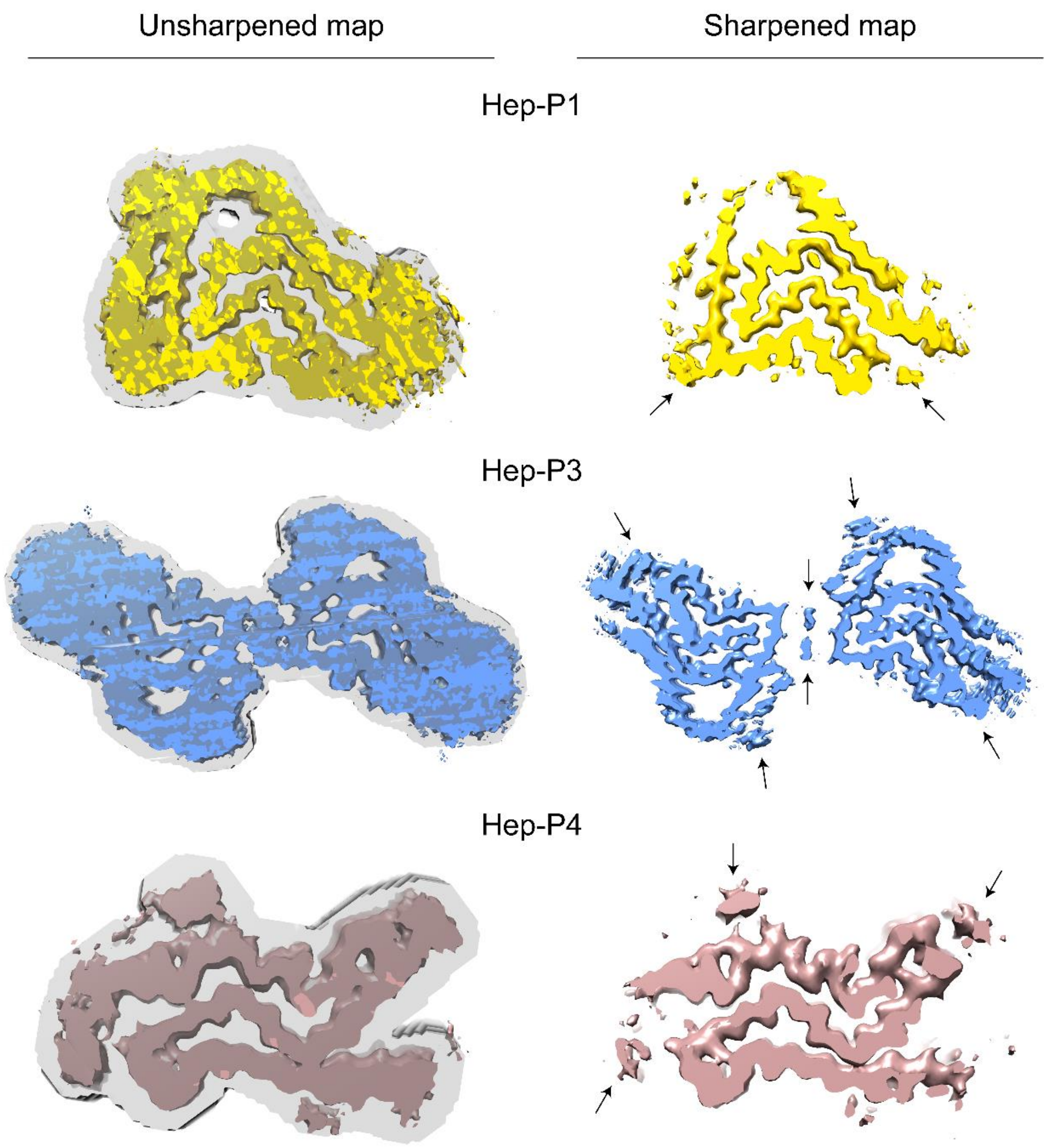

Supplementary Figure 4. Sharpening for heparin-α-syn fibril maps.
Unsharpened maps are shown on the left with initial binarization threshold of 0.0049 for unsharpened Hep-P1 map, 0.00214 for unsharpened Hep-P3 map, and 0.00578 for unsharpened Hep-P4 map, respectively. The masks used for post-processing are shown in gray.
After post-processing, sharpened maps are shown on the right with threshold of 0.0125 for sharpened Hep-P1 map, 0.008 for sharpened Hep-P3 map, and 0.0095 for sharpened Hep-P4 map, respectively. The extra densities corresponding to heparin are indicated by arrows.

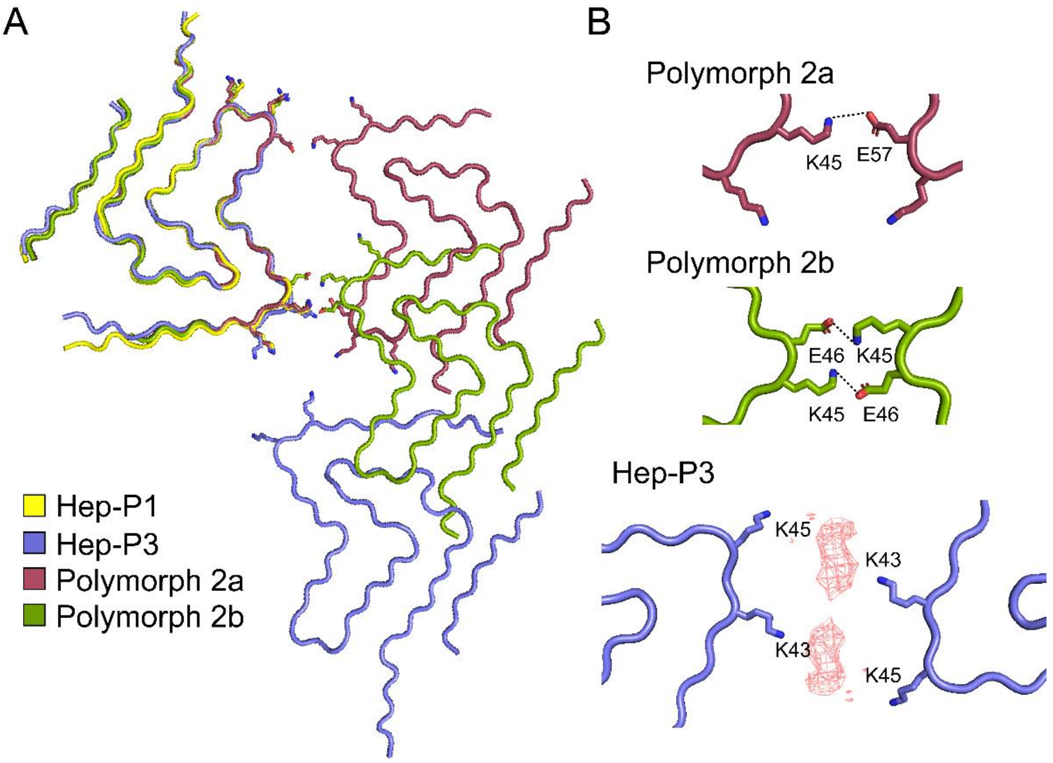

Supplementary Figure 5. Structural comparison of Hep-P1 with Hep-P3, α-syn fibril polymorphs 2a and 2b.

A. Alignment of the α-syn structures in one layer of the fibrils including Hep-P1 (yellow, monomer), Hep-P3 (blue, dimer), polymorph 2a (purple, dimer) and polymorph 2b (green, dimer). The root mean square deviations (RMSD) between Hep-P1 and Hep-P3 is 0.972 Å over 62 C-α atoms. The RMSD between Hep-P1 and polymorph 2a (PDB ID: 6SSX) is 0.961 Å over 63 C-α atoms. The RMSD between Hep-P1 and polymorph 2b (PDB ID: 6SST) is 0.688 Å over 60 C-α atoms.

B. Zoom-in views of the protofilamental interfaces of polymorph 2a, polymorph 2b and Hep-P3. Residues involved in the interfaces are highlighted and the side chains are shown as sticks. The pink meshes in Hep-P3 represent the densities of heparin.

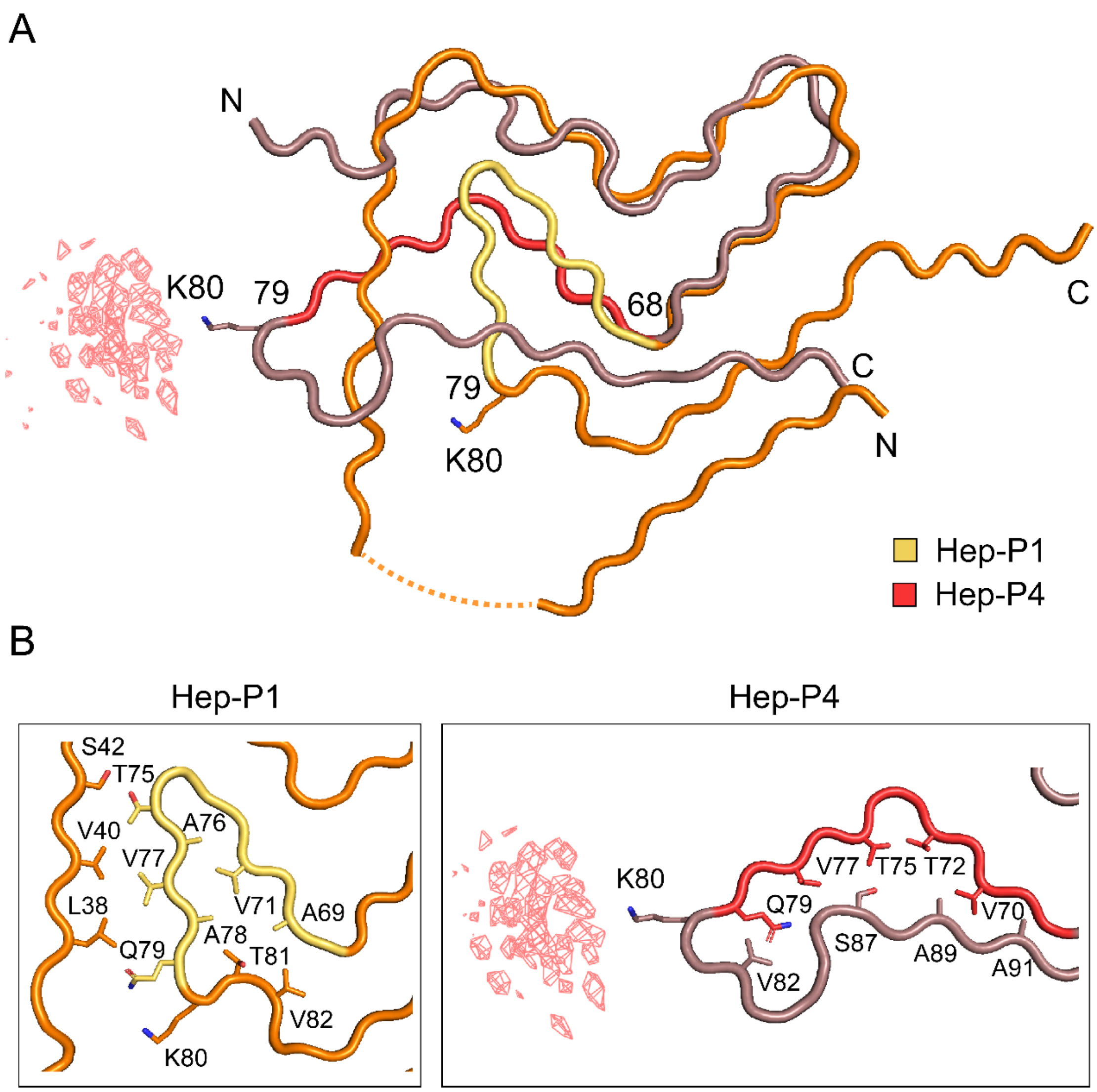

Supplementary Figure 6. Heparin binding with K80 drives the new fold generation in Hep-P4.

A. Overlay of the α-syn structures of Hep-P1 (brown) with Hep-P4 (yellow). The V-shaped structural motif formed by residues 68-79 are colored in orange (Hep-P1) and red (Hep-P4), respectively. The additional density adjacent to K80 in Hep-P4 is shown by mesh.

B. Zoom-in views of the local conformation of the V-shaped structural motif in Hep-P1 (left) and Hep-P4 (right). The side chains are shown as sticks.

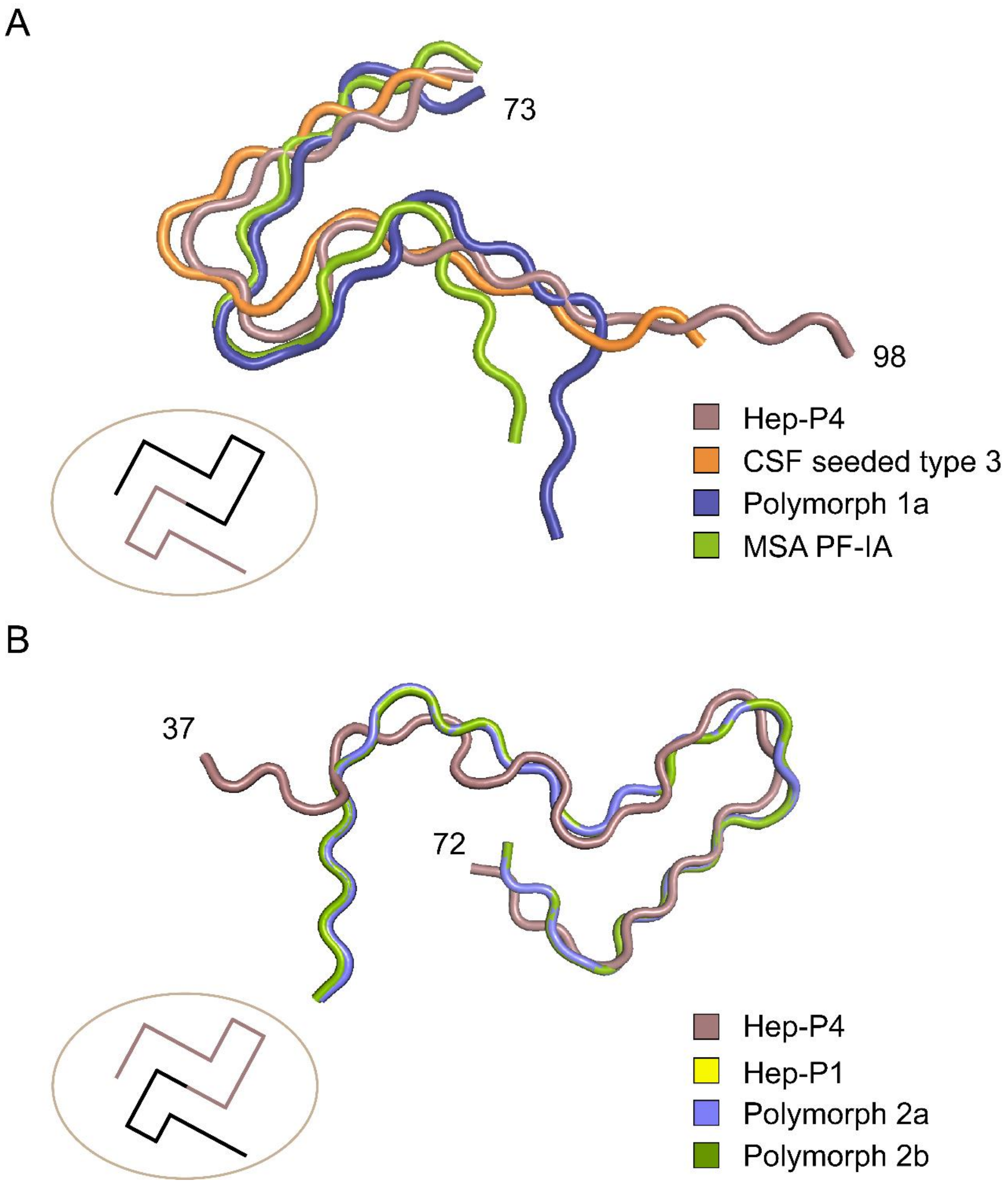

Supplementary Figure 7. Hep-P4 is a hybrid structure of two common α-syn structural motifs.

A. Structural comparison of the C-terminal half (residue 73-98) of Hep-P4 with CSF seeded type 3 polymorph (PDB ID: 7V49, RMSD 2.766 Å over 22 C-α atoms), polymorph 1a (PDB ID: 6A6B, RMSD 3.506 Å over 22 C-α atoms), and MSA PF-IA (PDB ID: 6XYO, RMSD 2.942 Å over 22 C-α atoms).

B. Structural comparison of the N-terminal half (residue 37-72) of Hep-P4 with Hep-P1 (RMSD 2.911 Å over 32 C-α atoms), polymorph 2a (PDB ID: 6SSX, RMSD 2.882 Å over 32 C-α atoms), and polymorph 2b (PDB ID: 6SST, RMSD 2.864 Å over 32 C-α atoms).

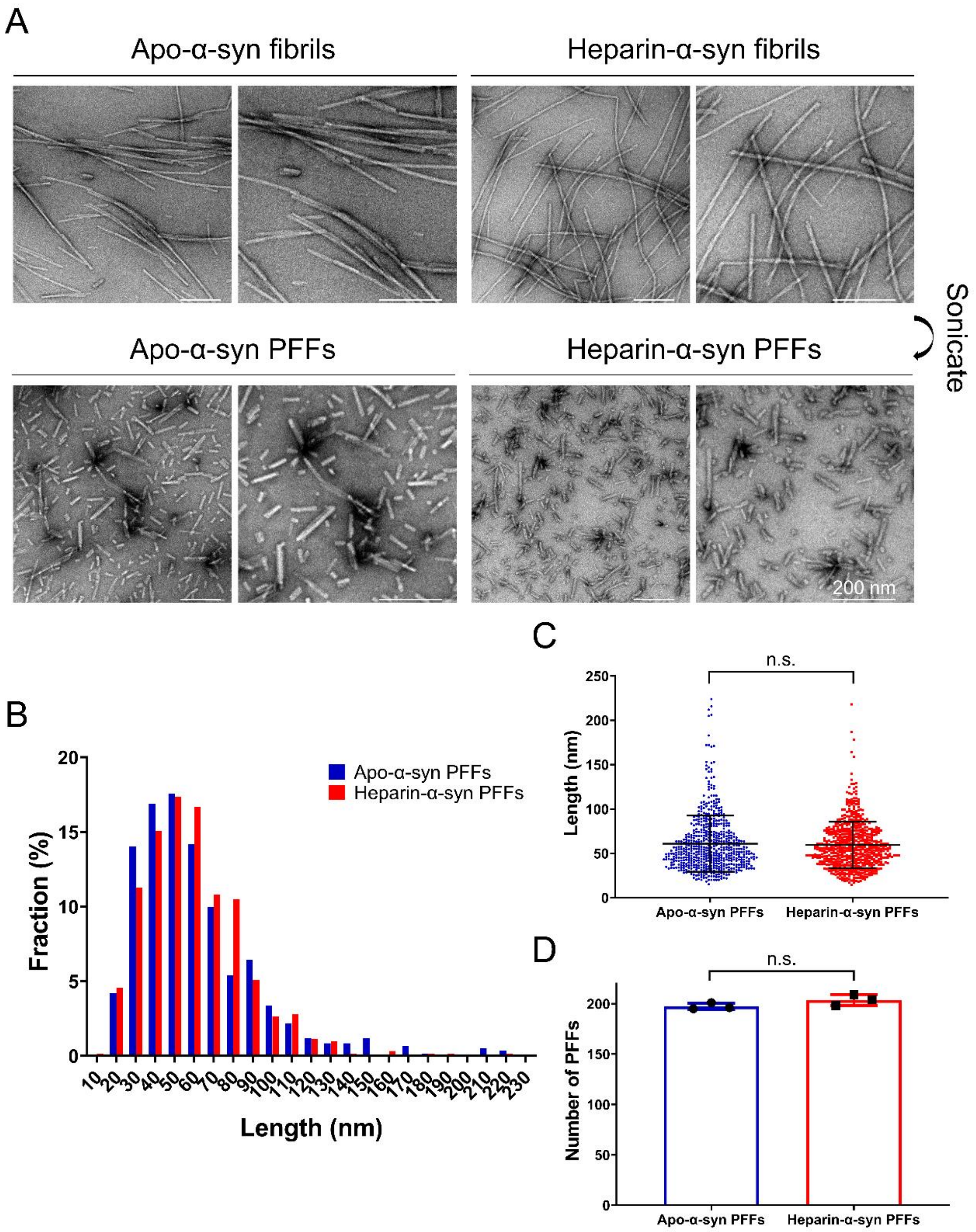

Supplementary Figure 8. Characterization and quantification of apo-α-syn PFFs and heparin-α-syn PFFs used in the cell assay.

A. Representative negative-staining TEM images of the apo-α-syn and the heparin-α-syn fibrils (top) and PFFs after sonication (bottom) from three biologically independent experiments. Images are shown with two magnifications for each sample. Scale bars are 200 nm.

B. Size distribution of apo-α-syn PFFs (592 particles) and heparin-α-syn fibrils PFFs (611 particles). The percentages for PFFs length were analyzed with Frequency distribution in GraphPad Prism 8.

C. PFFs length of apo-α-syn PFFs (592 particles) and heparin-α-syn PFFs (611

particles). Data are shown as mean ± s.d. The length of PFFs were measured from 3 independent reproducible TEM images. P=0.3723, unpaired, two-tailed Student's t test. **D.** Amounts of apo-α-syn PFFs and heparin-α-syn PFFs. The amounts were counted in 3 independent reproducible TEM images and shown as mean ± s.d. P=0.1605, unpaired, two-tailed Student's t test.

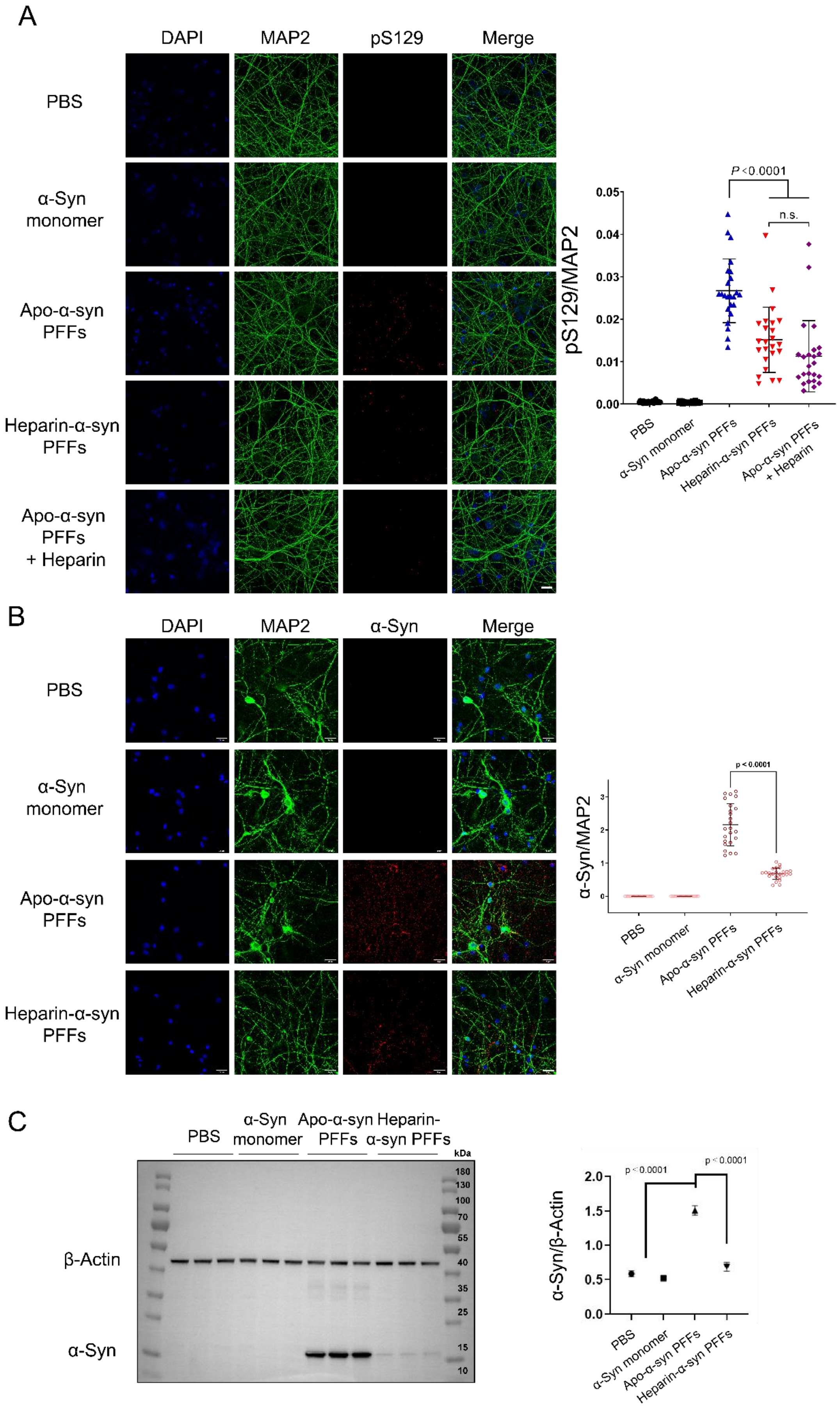

A
DAPI
MAP2
pS129
Merge
PBS
α-Syn monomer
Apo-α-syn PFFs
Heparin-α-syn PFFs
Apo-α-syn PFFs + Heparin
pS129/MAP2
P < 0.0001
n.s.
0.05
0.04
0.03
0.02
0.01
0.00
PBS
α-Syn monomer
Apo-α-syn PFFs
Heparin-α-syn PFFs
Apo-α-syn PFFs + Heparin
B
DAPI
MAP2
α-Syn
Merge
PBS
α-Syn monomer
Apo-α-syn PFFs
Heparin-α-syn PFFs
α-Syn/MAP2
p < 0.0001
3
2
1
0
PBS
α-Syn monomer
Apo-α-syn PFFs
Heparin-α-syn PFFs
C
PBS
α-Syn monomer
Apo-α-syn PFFs
Heparin-α-syn PFFs
kDa
180
130
100
70
55
40
35
25
15
10
β-Actin
α-Syn
α-Syn/β-Actin
p < 0.0001
p < 0.0001
2.0
1.5
1.0
0.5
0.0
PBS
α-Syn monomer
Apo-α-syn PFFs
Heparin-α-syn PFFs

Supplementary Figure 9. Heparin-α-syn complex fibrils show attenuated neuropathology.

A. Left: Representative immunofluorescence images of rat primary cortical neurons treated with PBS, 200 nM α-syn monomer, 200 nM apo-α-syn PFFs, 200 nM heparin-α-syn PFFs, and 200 nM apo-α-syn PFFs + 2.4 μg ml^{-1} heparin (comparable amount of heparin with that in heparin-α-syn PFFs, mixed in medium). The fixed neurons were immunostained for DAPI (blue), MAP2 (green), phosphorylated S129 α-syn (pS129, red). Scale bar, 20 μm. Right: Quantification of the pS129 intensity normalized to MAP2 intensity. Data are shown as mean ± SD of 24 images in three independent experiments. Statistical significance was measured using one-way ANOVA followed by Tukey's post-hoc test. There was no significance between heparin-α-syn PFFs group and apo-α-syn PFFs+heparin group (P=0.1835).

B. Left: Representative immunofluorescence images of the α-syn surface binding on rat primary cortical neurons treated with PBS, α-syn monomer, apo-α-syn PFFs and hep-α-syn PFFs. The fixed neurons were immunostained for DAPI (blue), MAP2 (green), α-syn (red). The α-syn antibody (ab138501) specifically recognizes human α-syn. Scale bar, 20 μm. Right: Quantification of the bound α-syn intensity normalized to MAP2 intensity. Data are shown as mean ± SD of 24 images in three independent experiments. Statistical significance was measured using one-way ANOVA followed by Tukey's post-hoc test.

C. Western blot of the α-syn that were taken up by primary cortical neurons. The α-syn antibody (ab138501) specifically recognizes human α-syn. β-Actin was used as a loading control. The cellular uptake of α-syn were quantitated by measuring the intensity of bands normalized to β-actin intensity. Data are means ± SD of three independent experiment samples. Statistical significance was measured using one-way ANOVA followed by Tukey's post-hoc test.

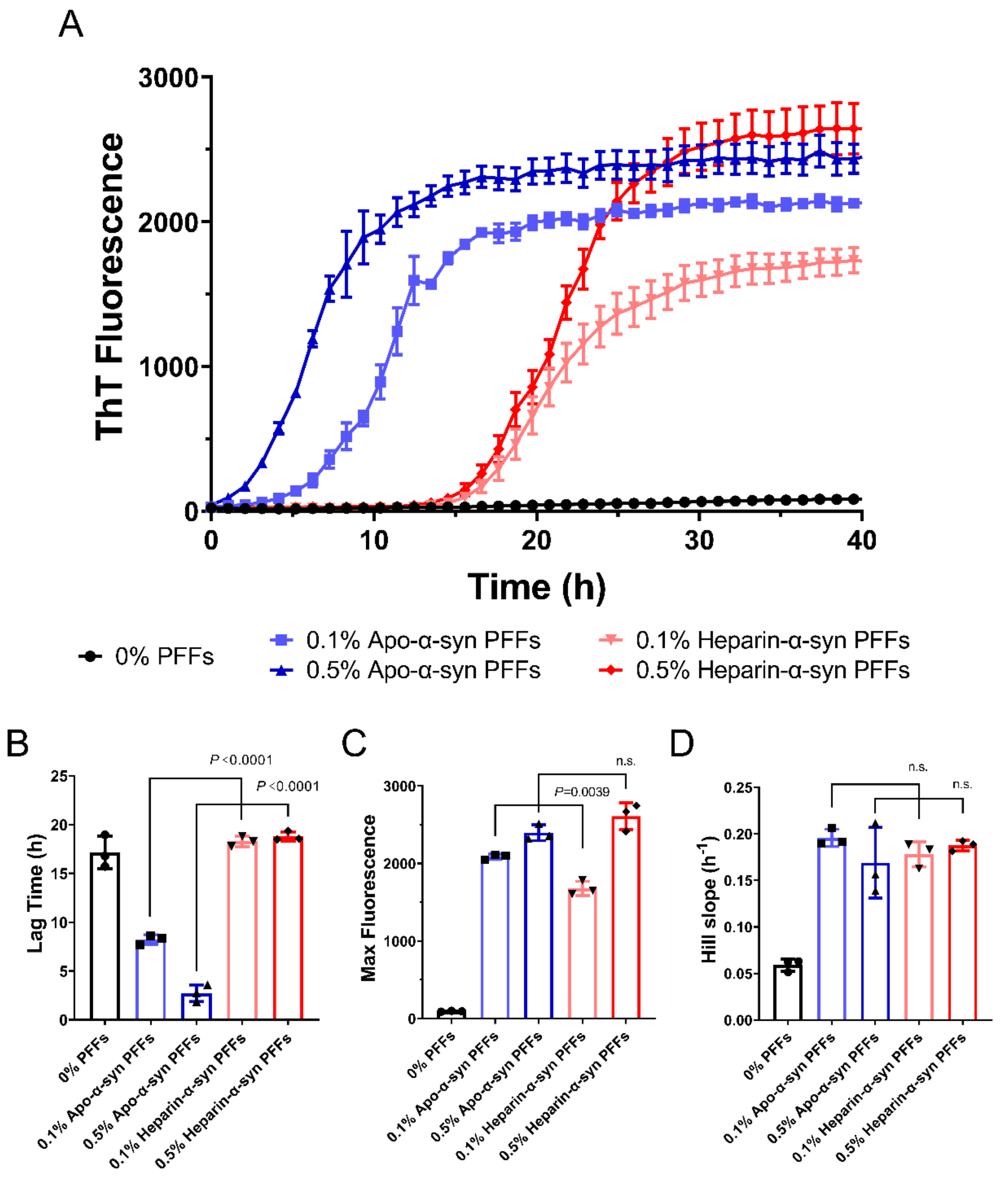

Supplementary Figure 10. Seeding properties of the apo-α-syn PFFs and heparin-α-syn PFFs.

A. ThT assay for α-syn alone (colored in black) or in the presence of 0.1 mol% apo-α-syn PFFs (colored in light blue), 0.5 mol% apo-α-syn PFFs (colored in dark blue), 0.1 mol% heparin-α-syn PFFs (colored in light red), and 0.5 mol% heparin-α-syn PFFs (colored in dark red), respectively. Data are shown as mean ± s.d., n = 3 independent samples.

B. The lag time of apo-α-syn PFFs seeded aggregation and heparin-α-syn PFFs seeded aggregation. Data are shown as mean ± s.d., n = 3 independent samples. Statistical significance was measured using one-way ANOVA followed by Tukey's post-hoc test.

C. The max fluorescence intensity of apo-α-syn PFFs seeded aggregation and heparin-α-syn PFFs seeded aggregation. Data are shown as mean ± s.d., n = 3 independent samples. Statistical significance was measured using one-way ANOVA followed by

Tukey's post-hoc test. There was no significance between 0.5 mol% apo-α-syn PFFs and 0.5 mol% heparin-α-syn PFFs group ($P=0.1476$).

D. The hill slope of apo-α-syn PFFs seeded aggregation and heparin-α-syn PFFs seeded aggregation. Data are shown as mean ± s.d., n = 3 independent samples. Statistical significance was measured using one-way ANOVA followed by Tukey's post-hoc test. There was no significance between apo-α-syn PFFs and heparin-α-syn PFFs group ($P=0.7799$ for 0.1 mol% seeds comparison, $P=0.7484$ for 0.5 mol% seeds comparison).

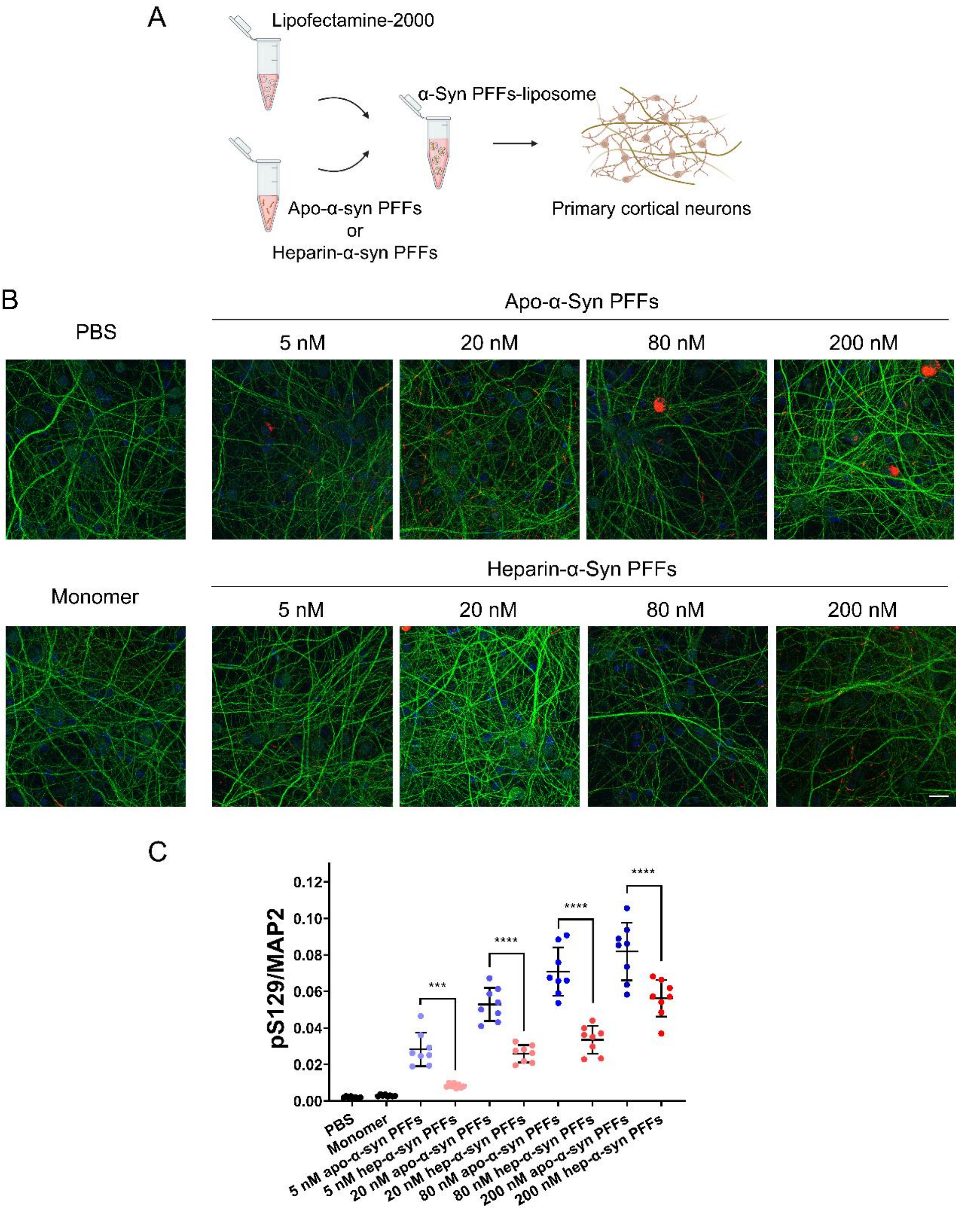

Supplementary Figure 11. Seeding capacity of apo-α-syn PFFs and heparin-α-syn PFFs to endogenous α-syn in primary neuron.

A. Schematic diagram of the liposome mediated seeds transduction process. The figure is created with BioRender.com.

B. Representative immunofluorescence images of rat primary cortical neurons treated with liposome with PBS, α-syn monomer, apo-α-syn PFFs (5, 20, 80, 200 nM), heparin-α-syn PFFs (5, 20, 80, 200 nM). The fixed neurons were immunostained for DAPI (blue), MAP2 (green), phosphorylated S129 α-syn (pS129, red).

C. Quantification of the pS129 intensity normalized to MAP2 intensity. Data are shown

as mean ± SD of 8 images in three independent experiments. Statistical significance was measured using one-way ANOVA followed by Tukey's post-hoc test. ***P=0.0009; ****P < 0.0001.
